# Polariton mediated electron transfer under the collective molecule–cavity coupling regime†

**DOI:** 10.1039/d5sc01911g

**Published:** 2025-05-19

**Authors:** Eric R. Koessler, Arkajit Mandal, Andrew J. Musser, Todd D. Krauss, Pengfei Huo

**Affiliations:** a Department of Chemistry, University of Rochester 120 Trustee Road Rochester NY 14627 USA ekoessle@ur.rochester.edu; b Department of Chemistry, Texas A&M University College Station TX 77842 USA; c Department of Chemistry and Chemical Biology, Cornell University Ithaca NY 14853 USA; d The Institute of Optics, Hajim School of Engineering, University of Rochester Rochester NY 14627 USA; e Center for Coherence and Quantum Optics, University of Rochester Rochester NY 14627 USA pengfei.huo@rochester.edu

## Abstract

We investigate polariton-mediated electron transfer (PMET) under the collective molecule–cavity coupling regime, with the presence of dark state transfer, cavity loss, and continuous-wave (CW) laser driving using quantum dynamics simulations and analytic rate constant theories. We demonstrate how the PMET rate constant can be enhanced by the collective coupling effect, where the light–matter coupling strength is small, yet there are many molecules collectively coupled to the cavity. We first show how reactions initialized in the collective upper polariton (UP) state can significantly enhance the PMET rate constant by decreasing the reaction driving force of an otherwise uphill ET reaction with collective strong coupling and positive detuning. We further show how the PMET rate constant is affected by dark states and cavity loss, which are often regarded as obstacles, and how to overcome them to provide a significant cavity-induced rate constant enhancement under the collective coupling regime. In particular, we show that by driving the UP state with a CW laser in a positively detuned cavity, the effective PMET rate constant can be several orders of magnitude larger than outside the cavity, even with significant molecular disorder and cavity loss. These results reveal a promising approach to realize photochemical rate enhancement with collective strong coupling in disordered and lossy polariton systems, as well as enabling otherwise impractical uphill ET reactions.

## Abstract 2

We investigate polariton-mediated electron transfer under the collective coupling regime in the presence of dark state transfer, cavity loss, and continuous-wave laser driving using quantum dynamics simulations and analytic rate theories.

## Graphical abstract

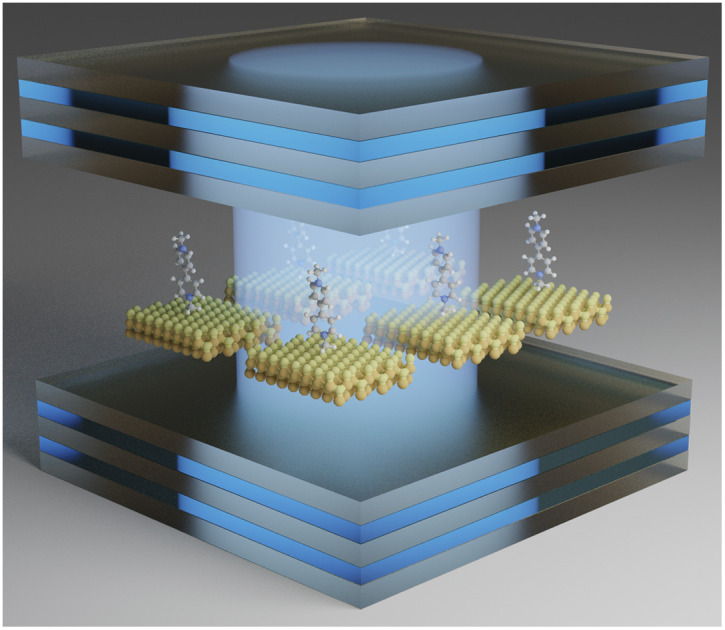

## 1 Introduction

The possibility of modifying and enhancing the rates of photochemical reactions by strong coupling to optical cavities has attracted the excitement of the polariton chemistry community in recent years.^1–5^ Following experimental demonstrations of chemical rate modification under the vibrational strong coupling (VSC) regime,^6–11^ recent works in the electronic strong coupling (ESC) regime have demonstrated the modification and enhancement of photochemical reactions inside optical cavities.^12–17^ While these recent polariton photochemistry experiments have yet to be fully understood with detailed microscopic theory,^18–20^ there have been numerous theoretical proposals that predict ESC photochemical and photophysical rate modifications inside the cavity for a variety of model systems, such as photoinduced charge transfer,^21–33^ singlet fission,^34–36^ intersystem crossing,^37,38^ exciton energy transfer,^39,40^ photodissociation,^41–49^ excited state proton transfer,^50^ and photoisomerization.^51–56^

While these recent experimental and theoretical polariton photochemistry works are exciting, there has also been concern expressed about the extent to which these proposed modifications can be realized when many (*N* ∼ 10^3^–10^9^) molecules are collectively coupled to the cavity.^20,57^ Some polariton photochemistry experiments in the collective coupling regime have failed to observe cavity rate modifications with theoretical explanations typically assigning blame to the presence of the dense manifold of the dark states or cavity loss.^17,58–60^ Many of the previous theoretical works predicting cavity modification of photochemistry used simplified model systems with only a single strongly-coupled molecule, a set of disorderless molecules, or assume a perfect lossless cavity for analytic and computational convenience and consequentially cannot address the issues of dark states and cavity loss.

Our work builds off of previous theoretical work on PMET,^21,22,26,29,31,61–63^ particularly the work of Mandal *et al.* in ref. 21, who performed explicit quantum dynamics simulations and described analytic Marcus theory rate constant expressions for PMET with a single molecule strongly coupled to a lossless cavity mode. Mauro *et al.* in ref. 22 described analytic Marcus and Fermi's golden rule (FGR) rate constant expressions for PMET with many identical molecules strongly coupled to a lossless cavity mode, as well as simulated PMET population dynamics with a kinetics model that included cavity loss and dark state transfer with FGR.

In this work, we investigate polariton-mediated electron transfer (PMET) in the collective coupling regime under the influence of dark states, cavity loss, and continuous-wave (CW) laser driving. We extend these previous works by performing explicit quantum dynamics simulations of many donor–acceptor molecules with donor states collectively coupled to a lossy cavity mode with CW laser driving. By focusing on the transfer from the polariton manifold of states to the excited acceptor states (*i.e*. without the influence of transfer to acceptor ground states), the monotonic population dynamics in this work (as opposed to the non-monotonic population dynamics observed in ref. 22) can be accurately fit with effective rate constants. Furthermore, the use of explicit quantum dynamics simulations in this work allows for accurate descriptions of bright polariton to dark state transfer rates in the disordered molecule regime. These effective rate constants fitted from explicit quantum dynamics simulations can describe the effects of cavity loss and dark state transfer on PMET in an intuitive fashion, which allows for a clear understanding of which parameter regimes and experimental approaches allow for significant PMET rate enhancement due to collective strong coupling.

We theoretically show how collective quantities, such as the Rabi splitting *Ω*_R_, manifest in the change of the PMET rate constant by rigorously deriving a Marcus-type rate constant. This rate theory suggests a possible mechanism that only depends on the 
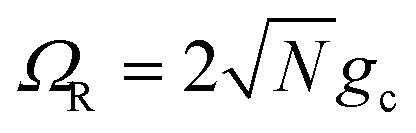
, even though the light–matter coupling strength per emitter *g*_c_ is small. We numerically verify the validity of this rate constant expression using the numerical simulations. Further, by driving the UP state with a CW laser in a positively detuned cavity, the effective PMET rate constant can be several orders of magnitude larger than outside the cavity even with significant molecular disorder (that causes transition to the dark states) and cavity loss (that causes population decay from UP to the ground state). These results reveal a promising mechanism to realize photochemical rate enhancement with collective strong coupling in disordered and lossy polariton systems, as well as enable otherwise impractical uphill ET reactions by using the higher energy of the UP. Note that uphill ET reactions could also be enhanced by coupling to the visible light field^64^ or by use a plasmonic effect.^65^ Here, our work demonstrates a promising principle based on polariton effects, and how delocalized light–matter interactions between one cavity mode and *N* molecules can induce changes in the rate constant for local chemical transformation.

## 2 Theory and model

### 2.1 Model Hamiltonian

**Fig. 1:**
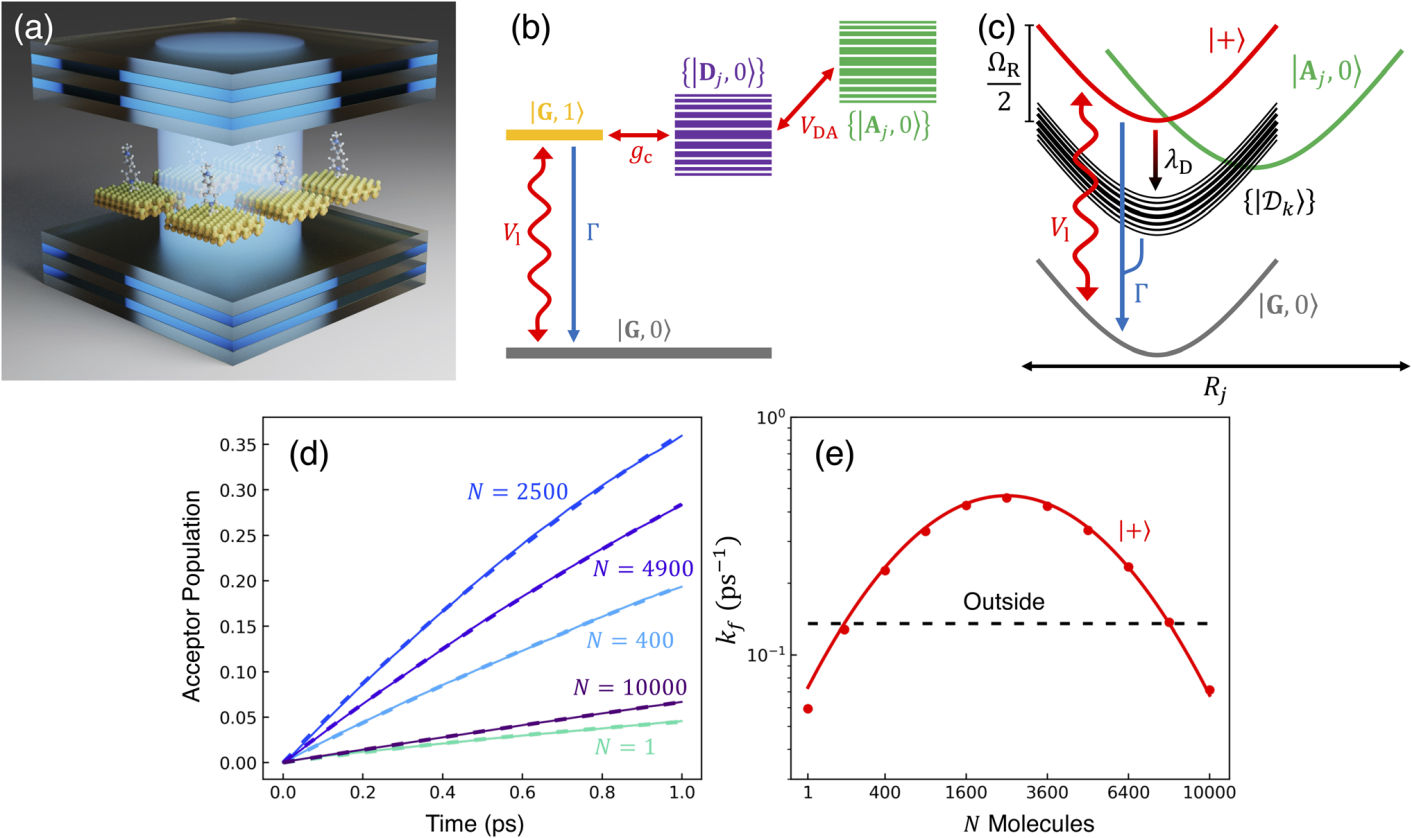
PMET model diagrams and collective rate enhancement from coupling many molecules with the cavity. (a) Illustration of several donor–acceptor pairs (visualized as CdSe nanoplatelets with attached methyl viologen) inside the cavity (visualized as a Fabry–Pérot cavity with distributed Bragg reflector mirrors). (b) Schematic energy levels of the system with couplings (red arrows) and incoherent cavity loss (blue arrow). (c) Schematic PESs along molecule *j*'s reaction coordinate *R*_*j*_ with population transfer pathways due to laser pumping (*V*_l_), cavity loss (*Γ*), and transfer from UP to dark states due to dynamical disorder induced by *λ*_D_. Note that here, we only visualize one of the states in the {|**A**_*j*_, 0〉} manifold, which contains a total of *N* acceptor states. (d) Acceptor population dynamics starting from the UP with *N* = 1, 400, 2500, 4900, and 10 000 molecules coupled to the cavity with a fixed *g*_c_ = 4 meV. The solid lines are the propagated populations, and the dashed lines are the populations based on the fitted rate constants. (e) Forward rate constants when starting from the UP inside the cavity (red) or a molecular state outside the cavity (black) for *N* ∈ [1, 10 000] molecules. The solid/dashed lines are the Marcus theory rates and the points are the fitted rates from the numerical propagation. Simulations were performed with *λ*_A_ = 200 meV, *V*_DA_ = 5 meV, Δ*G* = 0, and *V*_l_ = *Γ* = *λ*_D_ = 0.

A illustration of the model system is provided in Fig. 1a, and the schematic energy diagrams for the model system are depicted in panels (b) and (c). We consider a model photoinduced ET system that is coupled to a single-mode cavity at the Tavis–Cummings (TC) level of approximation. A relevant photoinduced ET system example is CdSe nanoplatelets (NPLs) as the photoinduced charge transfer donor molecules and organic molecules (such as viologen molecules) as the charge acceptor. The CdSe NPLs and viologen molecules outside the cavity have been previously synthesized and reported in the literature,^66^ and the photoinduced charge transfer kinetics have also been investigated. We consider each donor to be coupled to an acceptor molecule through chemically local electronic coupling *V*_DA_. We further consider that the cavity mode couples collectively to a total of *N* donor–acceptor molecule pairs, through the transition dipole of the donor (CdSe NPL), which has been accomplished in our recent experimental work.^67–70^ The light–matter interactions, as opposed to the electronic coupling, are highly non-local between the cavity mode and *N* molecules.

The total system Hamiltonian for the model is

1 *Ĥ* = *Ĥ*_m_ + *Ĥ*_p_ + *Ĥ*_mp_ + *Ĥ*_l_,

where *Ĥ*_m_ is the molecular Hamiltonian, *Ĥ*_p_ is the photonic Hamiltonian, *Ĥ*_mp_ describes the quantum light–matter interactions between molecules and photonic modes, and *Ĥ*_l_ describes the continuous-wave (CW) laser driving of the hybrid system.

The molecular Hamiltonian 
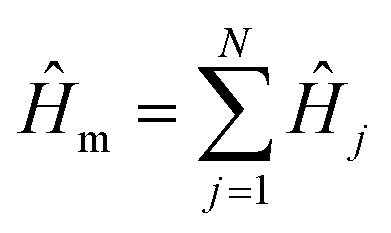
 describes a total of *N* non-interacting molecules. Each *Ĥ*_*j*_ contains three electronic states: ground state |G〉, donor state |D〉, and acceptor state |A〉, with an ET reaction coordinate *R* (Marcus coordinate^71^). The model Hamiltonian for a single donor–acceptor pair is^21^

2 *Ĥ*_*j*_ = ½*m*_S_*ω*_S_^2^*R*_j_^2^|G_*j*_〉〈G_*j*_| + (Δ*E*_D_ + ½*m*_S_*ω*_S_^2^(*R*_*j*_ − *R*^0^_D_)^2^)|D_*j*_〉〈D_*j*_| + (Δ*E*_A_ + ½*m*_S_*ω*_S_^2^(*R*_*j*_ − *R*^0^_A_)^2^)|A_*j*_〉〈A_*j*_| + *V*_DA_(|D_*j*_〉〈A_*j*_| + |A_*j*_〉〈D_*j*_|) + *T̂*_S,*j*_ + *Ĥ*_sb,*j*_,

where *m*_S_ and *ω*_S_ are the mass and frequency, respectively, of the reaction coordinate with position *R*_*j*_, Δ*E*_D_ and Δ*E*_A_ are the energy shifts of the donor and acceptor states, respectively, *R*^0^_D_ and *R*^0^_A_ are the minimum energy positions of the donor and acceptor states, respectively, with reorganization energies *λ*_D_ = ½*m*_S_*ω*_S_^2^*R*^0^_D_^2^ and *λ*_A_ = ½*m*_S_*ω*_S_^2^*R*^0^_A_^2^, *V*_DA_ is the diabatic coupling between the donor and acceptor, *T̂*_S,*j*_ = *P*_*j*_^2^/2*m*_S_ is the kinetic energy of the reaction coordinate with momentum *P*_*j*_, and *Ĥ*_sb,*j*_ is the Caldeira–Leggett system-bath interaction^72^ of the reaction coordinate with a phonon bath3
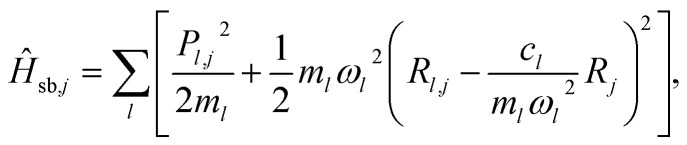
where *R*_*l*,*j*_ and *P*_*l*,*j*_ are the position and momentum, respectively, of bath mode *l*, and *ω*_*l*_ and *c*_*l*_ are the frequency and coupling strength, respectively, of bath mode *l* that are described with the ohmic spectral density *J*(*ω*) = *ζω* e^−*ω*/*ω*_0_^. The friction parameter *ζ* determines the overall system-bath coupling strength and the high frequency cut-off is *ω*_0_ ≫ *ω*_S_.

We focus on the single excitation subspace.^1,22^ This includes the collective ground state |**G**〉 and singly excited states |**D**_*j*_〉 and |**A**_*j*_〉 (where *j* ∈ [1, *N*] labels the molecules), defined as

4a |**G**〉 ≡ |G_1_〉 ⊗…⊗ |G_*N*_〉,

4b |**D**_*j*_〉 ≡ |G_1_〉 ⊗…⊗ |D_*j*_〉 ⊗…⊗ |G_*N*_〉,

4c |**A**_*j*_〉 ≡ |G_1_〉 ⊗…⊗ |A_*j*_〉 ⊗…⊗ |G_*N*_〉.

The photonic Hamiltonian of a single quantized cavity mode is described as

5 *Ĥ*_p_ = ℏ*ω*_c_(*â*^†^*â* + ½),

where *ω*_c_ is the cavity frequency and *â* is the annihilation operator of the cavity mode. The light–matter interaction Hamiltonian is described as6
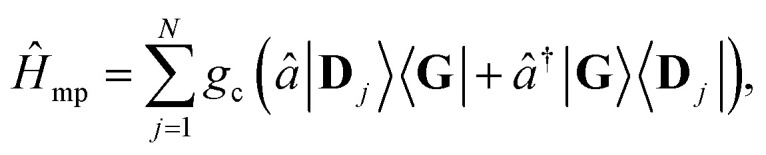
where *g*_c_ is the light–matter coupling strength between a single molecule and the cavity mode. This interaction is described at the Tavis–Cummings level of approximation^1,73–75^ which includes the rotating wave approximation and the lack of dipole self-energy, which are reasonable approximations in the coupling regime 
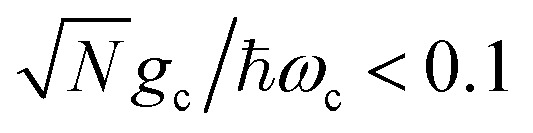
 (before entering into the ultra-strong coupling regime^21^). Additionally, each molecule's transition dipole is assumed to be aligned with the cavity field polarization and identical in magnitude such that each molecule's coupling *g*_c_ is identical and static.

The CW laser Hamiltonian *Ĥ*_l_ describes a continuous driving between the cavity states of the system^67^ and is expressed as

7 *Ĥ*_l_(*t*) = *V*_l_(*â* e^*iω*_l_*t*^ + *â*^†^ e^−*iω*_l_*t*^),

where *V*_l_ is the laser coupling strength and *ω*_l_ is the frequency of the laser. This laser model assumes the external laser is directly coupling to the cavity mode through a partially-reflective mirror or other cavity structure.^76^

In addition to the Hamiltonian dynamics, the effect of cavity loss is included in the system through the Lindblad master equation^77–79^8
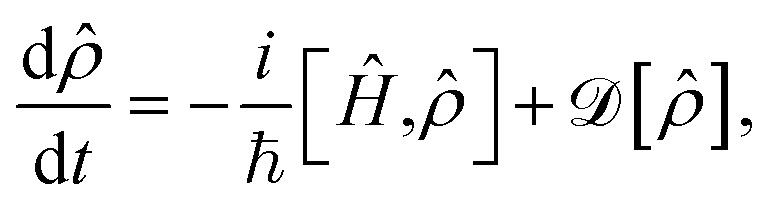
where 
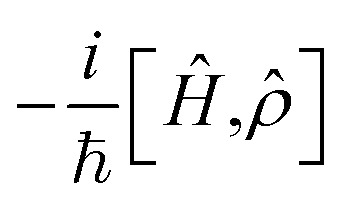
 describes the Hermitian evolution of the reduced density matrix *ρ̂* due to *Ĥ* and the dissipator 
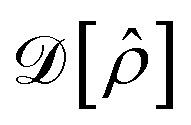
 is9
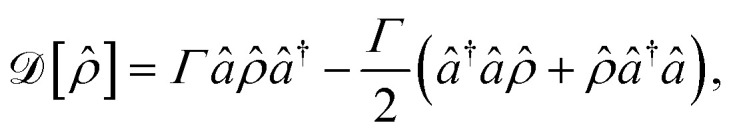
which causes the loss of cavity photons, where *Γ* is the cavity loss rate.

The cavity mode in the single excitation subspace is treated as a two-level system with states |0〉, which is the vacuum 0-photon Fock state whose energy is set to 0 for convenience, and |1〉, which is the 1-photon Fock state with energy *ℏω*_c_. In the combined molecule–cavity Hilbert space, which is restricted to zero and single-excitation states (see Fig. 1b), we label the states in a condensed fashion (*e.g.*, |**G**〉 ⊗ |1〉 ≡ |**G**, 1〉). For brevity, further labels of excited molecular states without a Fock state label are assumed to have 0 photons (*e.g.*, |**A**_*j*_〉 implies |**A**_*j*_, 0〉).

We further define the polaritonic Hamiltonian as10
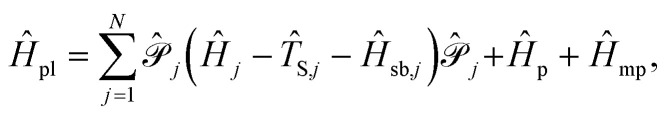
where 
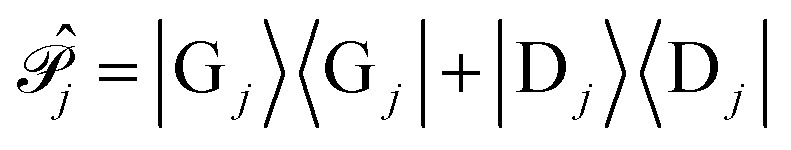
 is the projection operator that projects onto the ground and donor molecular states which excludes the acceptor states from *Ĥ*_pl_.

The eigenstates of *Ĥ*_pl_ are the so-called polariton states of the system. For a given nuclear configuration **R** ≡ {*R*_*j*_}, these polariton states |*ψ*_*n*_(**R**)〉 and their corresponding eigenenergies *E*_*n*_(**R**) can be determined by the following eigenvalue equation

11 *Ĥ*_pl_(**R**)|*ψ*_*n*_(**R**)〉 = *E*_*n*_(**R**)|*ψ*_*n*_(**R**)〉,

where *n* labels the eigenstates. The polariton states with a single excitation can be expanded in the diabatic-Fock basis as12
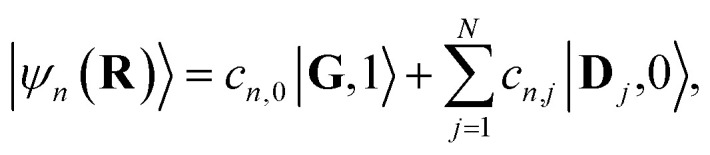
where *c*_*n*,0_ is the eigenstate expansion coefficient of the *n*th polariton state for |**G**, 1〉 and *c*_*n*,*j*_ is the eigenstate expansion coefficient of the *n*th polariton state for |**D**_*j*_, 0〉. For the case *λ*_D_ = 0, the donor state energies are identical for all **R** and consequentially the polariton states can be separated into bright and dark states. This also holds for the case *λ*_D_ ≠ 0 and **R** = **0** (more generally, when all *R*_*j*_ are identical). The bright polariton states are the upper polariton (UP) and lower polariton (LP) states13a
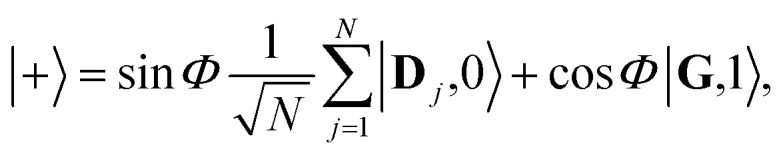
13b
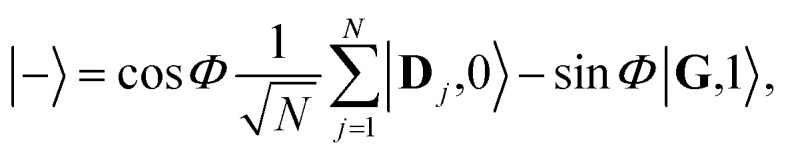
where |+〉 and |−〉 are the UP and LP states, respectively, and the mixing angle *Φ* is14
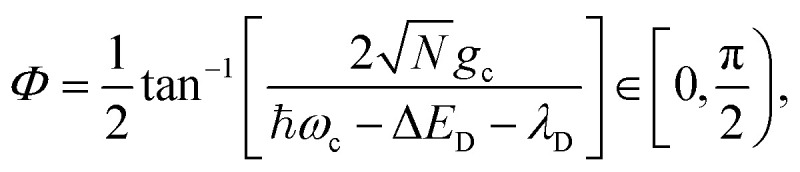
where the cavity detuning is *δ*_c_ = *ℏω*_c_ − Δ*E*_D_ − *λ*_D_. The energy difference between the UP and LP is known as the Rabi splitting, which can be expressed as15
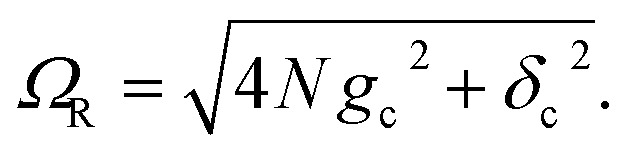
The remaining *N* − 1 eigenstates are the dark states 
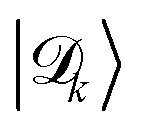
 (not to be confused with the donor exciton states |**D**_*j*_, 0〉) which are superpositions of only {|**D**_*j*_, 0〉} states that can be expressed as follows^1,75,80^16
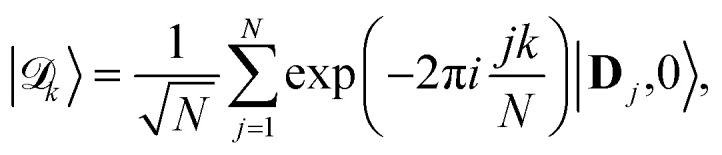
where *k* ∈ {1, …, *N* − 1} labels the dark states. The energy of the dark states remains the same as the exciton site energy, but with a large density of states *N* − 1. Note that the dark states have no overlap with the collective “bright” states, and they do not participate in the interaction with the cavity mode mediated by *Ĥ*_mp_ since 
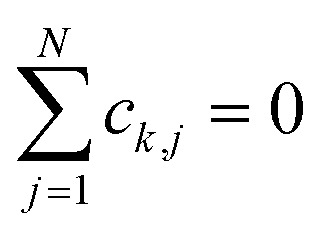
 which renders them optically dark (*i.e.* having no transition dipole from the ground state |**G**〉). As a reminder, the above definitions of the bright polariton and dark states do not have explicit static disorder taken into account. Generally, static disorder can significantly impact polariton dynamics^81^ and polariton delocalization.^82^ Our bath model in this work is equivalent to a Brownian oscillator spectral density^83^ with a peak frequency at *ℏω*_S_ ≈ 9.54 meV thus most of the effective phonon modes are low frequency and can be expected to behave similarly to explicit static disorder with a standard deviation of^84^
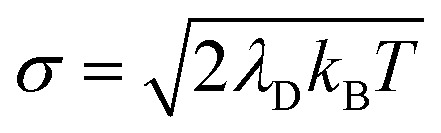
.

When the cavity is in resonance with the donor energy (*Φ* = π/4), the UP and LP states without disorder simplify to17
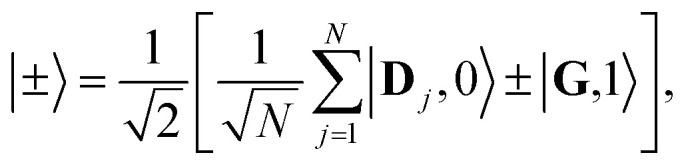
such that the UP and LP eigenstates are half donor character (equally distributed among all *N* molecules) and half photonic character. The Rabi splitting at resonance is 
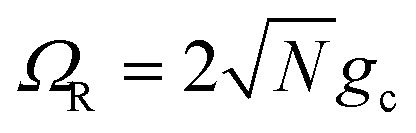
. For *λ*_D_ ≠ 0, the donor state energies may not be identical (*i.e.* disordered) for **R** ≠ **0** which causes the polariton eigenstates to no longer be perfectly bright or dark.^68,85,86^ In particular, there may be quasi-dark eigenstates^67^ with small amounts of photonic character, as well as eigenstates similar to the disorderless UP and LP states but with different amounts of donor character for different molecules.^67^

### 2.2 Origin of the collective effect in PMET

Here, we develop an analytic theory to describe the collective PMET rate constant. The details of the derivations are provided in the ESI.† For a reaction occurring across a large number of nuclear coordinates **R** among a set of electronic–photonic states, a collective reaction coordinate **R**^RC^_*ab*_(*X*) from state |*a*〉 to state |*b*〉 can be defined as a function of the energy difference *X*. For a fixed *X* = Δ*E*_*ab*_(**R**), the collective reaction coordinate **R**^RC^_*ab*_(*X*) is the nuclear configuration that minimizes the donor energy *E*_*a*_(**R**).^87^ This collective reaction coordinate can be used to derive reaction parameters such as the driving force and reorganization energy.

For a collective PMET reaction from the UP state |+〉 with mixing angle *Φ* to an individual acceptor state |**A**_*j*_〉, the collective reaction coordinate can be shown to be18
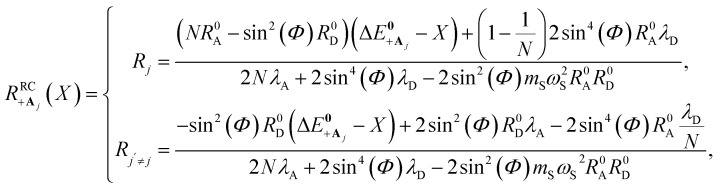
where 
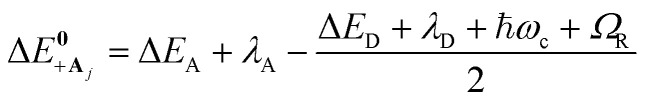
 is the energy difference between states |**A**_*j*_〉 and |+〉 at **R** = **0** (see the ESI† for a detailed derivation). In the collective limit *N* → ∞ (with a fixed collective light–matter coupling 
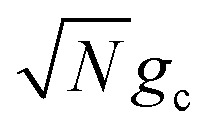
), the collective reaction coordinate simplifies to be19
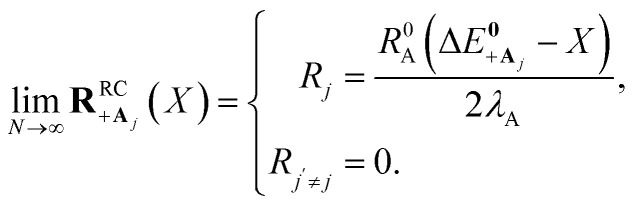
This demonstrates that in the collective limit, PMET reactions from the UP to the acceptor state of the *j*th molecule only depend on the PESs along the reaction coordinate of the *j*th molecule.^52,54,58,74^

To determine reaction parameters such as the reorganization energy and the driving force, the energy differences 
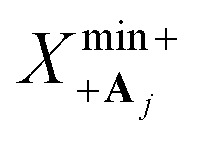
 and 
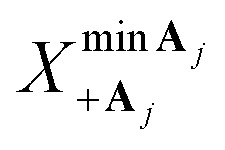
 that minimize the energies of states |+〉 and |**A**_*j*_〉, respectively, along 
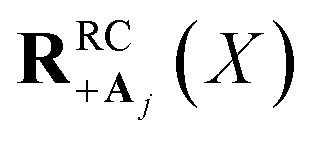
 can be used. The corresponding reorganization energy *λ*_+**A**_*j*__ between states |+〉 and |**A**_*j*_〉 is20a
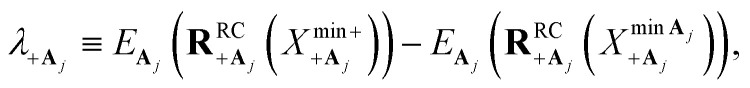
20b
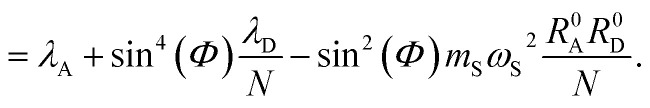


The polaron decoupling effect^24^ of the |+〉 state can be seen in the collective limit 
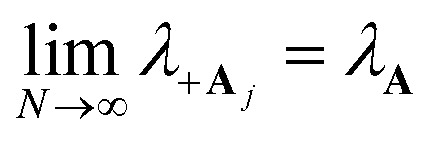
 where the contribution of the |+〉 state to the reorganization energy goes to 0. A similar calculation shows that the reorganization energy between the collective ground state |**G**, 0〉 and |+〉 is21a
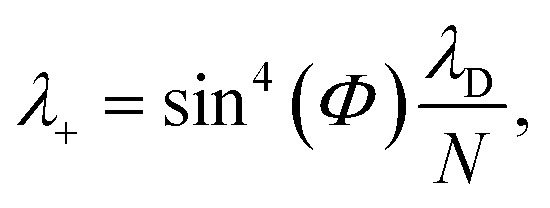
21b
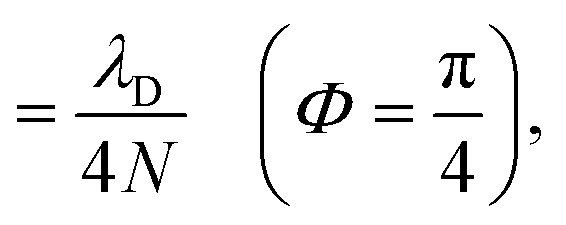
which is consistent with previous descriptions of the polaron decoupling of the |+〉 state.^1,24,75,88^ Interestingly, the expression of *λ*_+**A**_*j*__ is similar to 
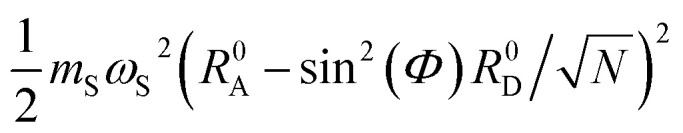
, which would be expected based on identifying 
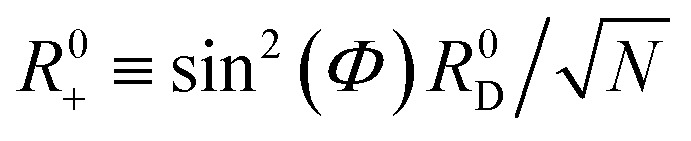
 as the collective nuclear displacement of the |+〉 state;^24^ however, the difference is that the cross-term in *λ*_+**A**_*j*__ has an extra factor of 
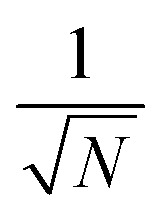
 compared to the cross-term in the expression ½*m*_S_*ω*_S_^2^(*R*^0^_A_ − *R*^0^_+_)^2^. Regardless, this difference becomes negligible in the collective limit.

The driving force Δ*G*_+**A**_*j*__ between states |+〉 and |**A**_*j*_〉 is22a
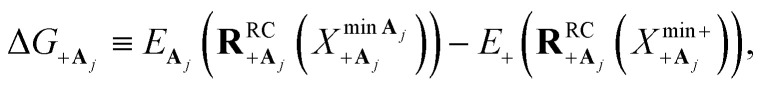
22b
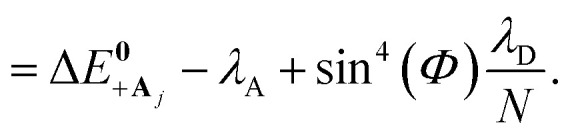


The collective limit of the driving force is23a
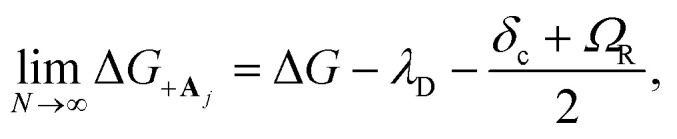
23b
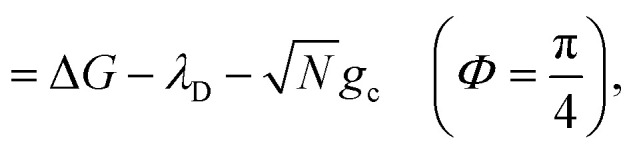
where Δ*G* = Δ*E*_A_ − Δ*E*_D_ is the bare driving force outside the cavity. This result confirms that the effect of modifying the driving force between the |+〉 and |**A**_*j*_〉 states by changing the collective light–matter coupling strength 
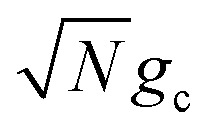
, which is observed in the single molecule case,^21^ is also seen in the collective limit.^22^ In addition, the driving force is modified by the detuning, where positive detuning decreases the driving force (decreasing the value of Δ*G*_+**A**_*j*__).

Another consequence of the result in eqn (23a) and (23b) is that the driving force is affected by the donor reorganization energy *λ*_D_ even in the collective limit such that there is no complete polaron decoupling effect for the driving force. At resonance, this is due to the increase in the minimum energy of the |+〉 state by *λ*_D_ which reduces the driving force by *λ*_D_.

### 2.3 Collective PMET rates

In the collective limit, the reorganization energy of the |+〉 state relative to the collective ground state |**G**, 0〉 is 
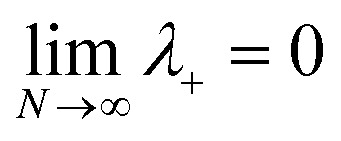
 which implies the |+〉 state has the same equilibrium geometry as |**G**, 0〉 (given that |**G**, 0〉 has a unique equilibrium geometry). This allows for equilibrium rates from the |+〉 state to an acceptor state to be calculated when the reaction is initiated with an FC excitation.

The traditional Marcus theory of nonadiabatic ET^71,87,89–91^ describes the reaction rate between a donor and acceptor state assuming a weak donor–acceptor coupling and a reaction initiated in the thermal equilibrium geometries of the donor state. The general Marcus rate constant expression is24
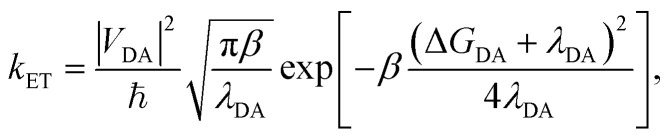
where *β* = 1/*k*_B_*T* with Boltzmann constant *k*_B_ and temperature *T*. The parameter regime where Marcus theory is accurate can more generally be described by the unitless adiabatic parameter^92,93^25
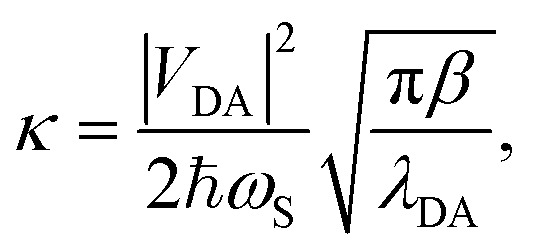
such that Marcus theory is accurate when *κ* ≪ 1 (and when the nuclei can be treated semi-classically under the condition *ℏω*_S_ < *k*_B_*T*). In the collective limit and for *κ* ≪ 1, the Marcus rate constant between states |+〉 and |**A**_*j*_〉 is26
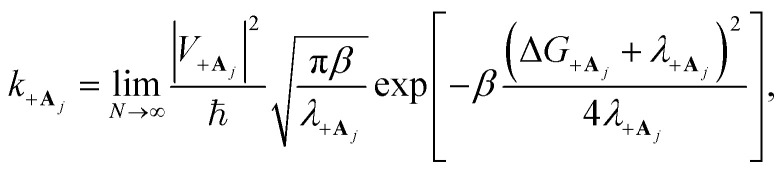
where the coupling between states |+〉 and |**A**_*j*_〉 is27
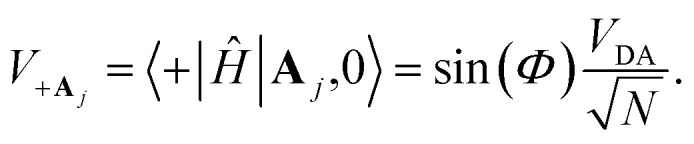
Note that there is a 
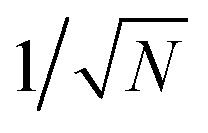
 “dilution” factor (normalization factor) in *V*_+**A**_*j*__. For an FGR type of estimation of rate constant, this results in a 1/*N* normalization factor, which often causes difficulties in theoretically observing any collective modification for polariton photophysics dynamics^59^ or in VSC rate constant changes.^63,94,95^ Nevertheless, the collective PMET rate can naturally avoid such a pitfall by considering the total rate constant 
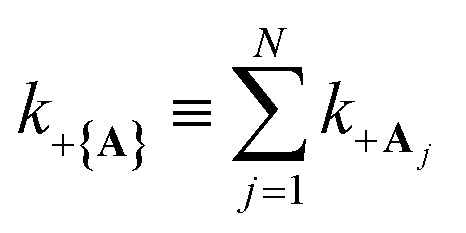
 between the |+〉 state and all possible acceptor states {|**A**_*j*_〉}, which can be can be expressed as28
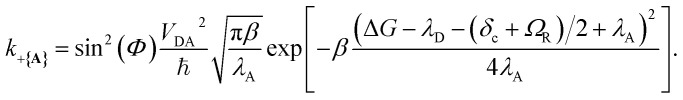
Importantly, the prefactor in *k*_+{**A**}_ is independent of *N* due to the cancellation of the 1/*N* factor in |*V*_+**A**_*j*__|^2^ with the sum over *N* acceptor states.^1,22^ The only *N* dependence in *k*_+{**A**}_ is the collective coupling 
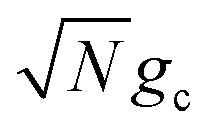
 which reduces the driving force (and alters the mixing angle *Φ* when detuning is present). For the case *λ*_D_ = 0, this total rate constant *k*_+{**A**}_ is equivalent to the Marcus PMET rate constant between the UP and acceptor for a single molecule with a light–matter coupling strength equal to 
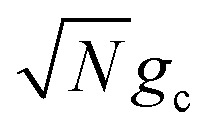
.^21^ Note that for the case *λ*_D_ ≠ 0, the disorderless |+〉 state defined in eqn (13a) is not an exact eigenstate of *Ĥ*_pl_ but may instead be an approximation of an eigenstate whose expansion coefficients are similar to that of |+〉 but whose acceptor couplings may differ from *V*_+**A**_j__ at 
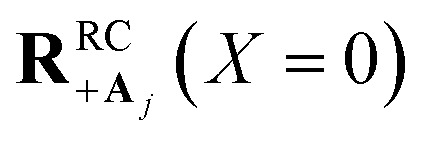
 and whose driving force may differ from Δ*G*_+**A**_*j*__. For a thermal Boltzmann distribution of nuclear positions, this approximation should be reasonable when the donor energy disorder^84^
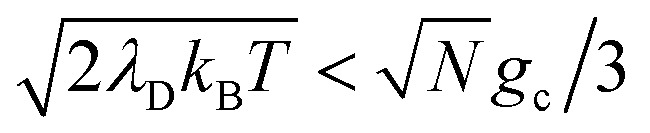
 (low molecular disorder relative to collective coupling strength^1,60^) and 
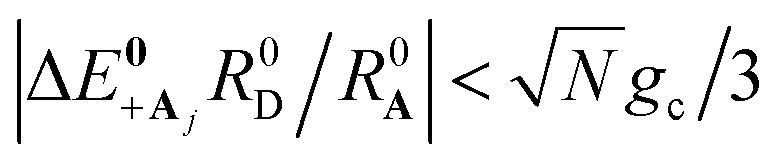
 (small detuning of donor state energy *E*_**D**_*j*__*versus E*_**D**_*j*′≠*j*__ at 
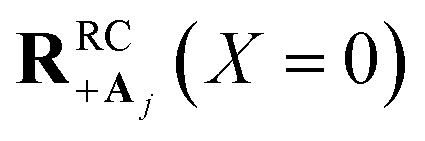
 relative to collective coupling strength to ensure the UP eigenstate has significant character |*c*_+,*j*_|^2^ ≈ sin^2^(*Φ*)/*N* of state |**D**_*j*_〉 in order to couple to state |**A**_*j*_〉 at 
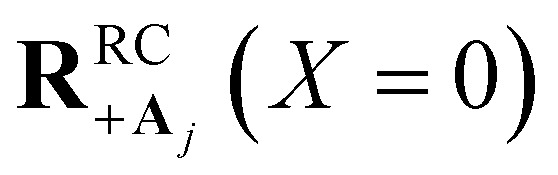
).

As shown in Fig. 1c, when any of the parameters *λ*_D_, *Γ*, or *V*_l_ are non-zero the dynamics of the polariton and acceptor states are affected by factors beyond those of the PMET rates in eqn (28). More specifically, a non-zero *λ*_D_ can allow for dynamical disorder among the polariton states which causes population transfer^58,67,68,74,80,85,96–101^ between the UP, LP, and dark states; a non-zero value of *Γ* causes population decay from photonic polariton states to |**G**, 0〉; and *V*_l_ causes population transfer between |**G**, 0〉 and photonic polariton states. While these factors create challenges for describing the population dynamics of the polariton and acceptor states with analytic expressions, explicit quantum dynamics simulations of the molecular–cavity system can provide these population dynamics beyond the limitations of analytic expressions. In this work, we calculate effective rate constants between the donor manifold of states (|**G**, 0〉 and the polariton states) and the acceptor manifold of states ({|**A**_*j*_〉}) by fitting the simulated explicit population dynamics of the acceptor states to a simple two-state ET model with fitted forward and backward rate constants. In particular, for a system initialized among the donor manifold of states, we fit the simulated population dynamics of the acceptor states 
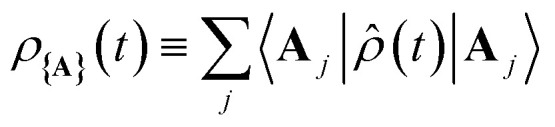
 to the following equation29
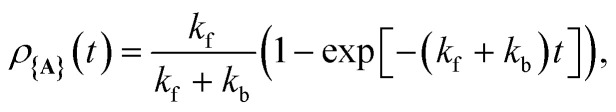
where *k*_f_ and *k*_b_ are the fitted effective forward and backward rate constants, respectively, between the donor and acceptor manifolds. For simulations initialized in the UP with *λ*_D_ = *Γ* = *V*_l_ = 0 and *κ* ≪ 1, the forward *k*_f_ fitted from short-time simulations should be nearly identical to the Marcus regime *k*_+{**A**}_ in eqn (28). Beyond this regime, the fitted rate constants may be significantly affected by the dynamics of dark state transfer, cavity loss, and laser driving. By calculating *k*_f_ from simulations, we can quantify in a simple manner the effects that these factors have on the effective transfer rates from the donor manifold to the acceptor manifold and thus their effect on PMET.

### 2.4 Simulation details

The reduced density matrix dynamics *ρ̂*(*t*) of the molecule–cavity PMET system in this work were propagated at the wavefunction level using the Lindblad mean-field Ehrenfest 
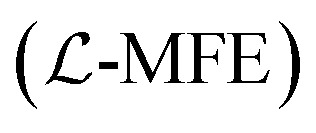
 approach.^79^ MFE is a mixed quantum-classical dynamics method that propagates a quantum subsystem (electrons and photons) alongside a classical subsystem (nuclei) which feel a mean-field force based on the quantum state.^102–104^ MFE has been shown to be accurate for model systems in the non-adiabatic regime (*κ* ≪ 1), including ET and other charge transfer processes.^21^
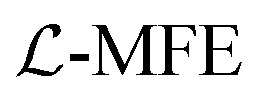
 incorporates Lindblad decay dynamics into MFE at the wavefunction level which allows for efficient and accurate simulation of cavity loss^68,70,79,105^ even in the presence of coherent laser pumping.

The state-independent phonon bath, described by *Ĥ*_sb,*j*_, was treated implicitly using a Langevin dynamics approach, which is equivalent to the reduced dynamics of the harmonic ET coordinate under the influence of an ohmic bath in the Markovian regime.^21,83,106,107^ This Langevin description reduces the computational cost of the many-molecule PMET simulation by reducing the number of propagated nuclear DOFs to one per molecule^91,108,109^ (see the ESI† for details). The computational cost of the simulation was further reduced by manually coding the result of the matrix–vector operation of *Ĥ* acting on the wavefunction (by taking advantage of the symmetry of the TC type Hamiltonian) in a way that scales linearly with *N*, see details in ref. 110. These approaches to reduce the computational cost allowed for *N* = 1000 molecules to be efficiently simulated on the nanosecond timescale. The number of trajectories was *N*_traj_ = 10, 000/*N* such that 10 trajectories were averaged over for the *N* = 1000 molecule simulations. This small number of trajectories required to reach convergence was possible due to the self-averaging of the population dynamics of the UP and the sum of the acceptor states when multiple molecules are coupled to the cavity. The nuclear time step was d*t*_N_ = 0.125 fs and the electronic time step was d*t*_E_ = d*t*_N_/4. The friction parameter used in this work was *ζ* = 1.066 × 10^6^ au and the mass was *m*_S_ = 4.529 × 10^8^ au for consistency with previous works on ET.^21,106^

## 3 Results and discussions

In this section, we explore the population dynamics and rates of our PMET model system in the collective coupling regime and compare them to the dynamics and rates outside the cavity. In all cases inside the cavity, we focus on cavities in resonance with the donor excited state in the FC configuration (*ℏω*_c_ = Δ*E*_D_ + *λ*_D_ = 3 eV) and reactions initiated in, or coherently pumped to, the UP state. This regime of focus allows us to examine the extent to which PMET reaction rates can be significantly enhanced, particularly in the presence of dark state transfer and cavity loss.

Fig. 1d and e present PMET in the Marcus regime for varying numbers of coupled molecules inside the cavity. The reaction inside the cavity is initiated as an FC excitation to the resonant |+〉 state. The ET parameters are *λ*_A_ = 200 meV, *V*_DA_ = 5 meV, and Δ*G* = 0 at room temperature *T* = 300 K. The nonadiabatic parameter for a single donor–acceptor pair is *κ* ≈ 0.032 ≪ 1 with *ℏω*_S_ ≈ 9.54 meV < *k*_B_*T* ≈ 25.8 meV and *λ*_D_ = *Γ* = *V*_l_ = 0 which confirms the dynamics are in the Marcus regime. Fig. 1d shows the propagated and fitted acceptor state populations for a variety of *N* with a fixed *g*_c_ = 4 meV. The system was propagated for 1 ps and *k*_f_ and *k*_*b*_ were fitted based on eqn (29) with the corresponding fitted populations from the right-hand side of eqn (29) plotted as dashed lines. The dynamics show a strong, non-monotonic dependence on *N* that accumulates acceptor population most rapidly near *N* = 2500 with reduced acceptor populations for larger or smaller *N*. Notably, the fitted populations show excellent agreement with the propagated populations which demonstrates that the short-time acceptor dynamics are well-captured by a 2-state rate constant model even when thousands of molecules are coupled to the cavity.

Fig. 1e shows the corresponding analytic Marcus forward rate constants and fitted forward rate constants based on the explicit quantum dynamics propagation. The Marcus rate constants based on eqn (28) are plotted (solid red line) over a continuous range of *N* ∈ [1, 10 000] molecules while the fitted rate constants (red dots) are plotted based on simulations for various *N* in the same range. The Marcus rate constant for a single donor–acceptor pair outside the cavity is also shown for comparison (black dashed line). The Marcus and fitted rate constants show excellent agreement, especially for *N* ≫ 1. The rate constant dependence on *N* inside the cavity appears as an inverted parabola (when the *N* axis scales as 
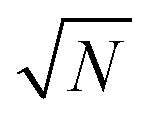
 and the *k*_f_ axis scales logarithmically) due to the collective coupling 
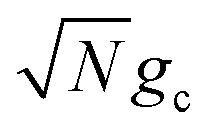
 effect on the driving force Δ*G*_+**A**_*j*__ (eqn (23b)). From *N* = 1 to *N* = 2500, the UP energy increases with *N* which decreases 
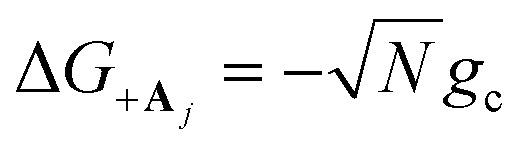
 and increases the rate constant until the reaction is barrier-less and the rate is maximized at *N* = 2500. For *N* > 2500, the UP energy continues to increase with *N* but the reaction is now in the inverted Marcus regime which decreases the rate as Δ*G*_+**A**_*j*__ becomes more negative. For 100 < *N* < 8100, the reaction inside the cavity is enhanced by up to a factor of 4 relative to outside the cavity due to the more favorable driving force Δ*G*_+**A**_*j*__ compared to the bare Δ*G* = 0 outside the cavity. Although the purpose of this panel is to validate our theory and the accuracy of our quantum dynamics simulations, it is nevertheless interesting to show one can selectively achieve the Marcus normal, activation less, and inverted regime with the same type of molecules by only varying *N*. Note that due to the large transition dipole of the CdSe NPL, we estimate that *N* = 10^3^–10^4^ NPLs coupled to the cavity can generate *Ω*_R_ ∼ 10^2^ meV Rabi splitting.^67,69,70^

It should be emphasized that the mechanism of enhancement in PMET demonstrated in Fig. 1 based on modifying the driving force relies on initiating the system in the UP state, which is a quantum state whose particular combination of energy and donor character does not exist outside the cavity near the FC region. A different mechanism for collective cavity-enhanced photochemistry was described in ref. 20 and 60 where the cavity filtered an initial FC cavity excitation into donor states with higher energy vibrational states during a series of early-time Rabi oscillations, where the higher energy vibrations allowed faster transfer to the acceptor state. This filtering mechanism, described for *T* = 0, relied on a non-zero *λ*_D_ and the observed enhancement vanished for *λ*_D_ = 0. In contrast, the enhancement mechanism in Fig. 1 based on increasing the energy of the electronic–photonic UP state using collective coupling does not fundamentally depend on filtering the donor vibrational states and does not diminish at *λ*_D_ = 0.

**Fig. 2:**
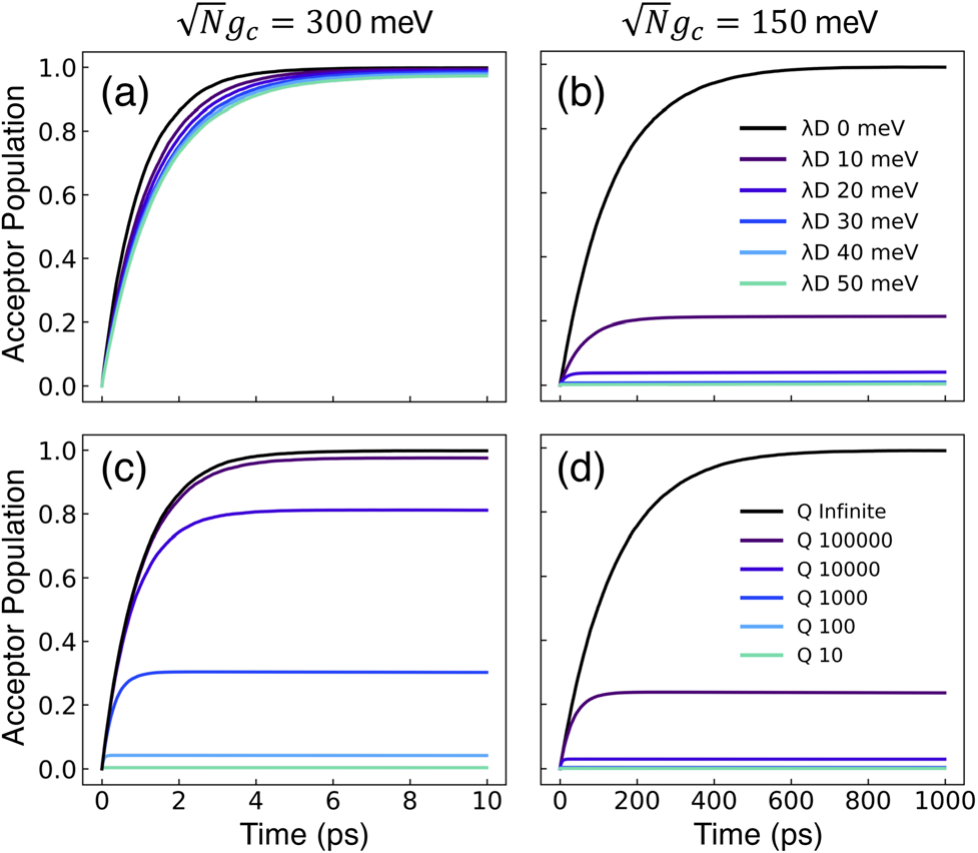
Effect of dark state transfer and cavity loss on acceptor populations. Panels (a) and (b) vary the donor reorganization energy *λ*_D_, which causes population transfer from the UP to the dark states. Panels (c) and (d) vary the quality factor *Q*, which causes population transfer from the UP to the ground state |**G**, 0〉. Panels (a) and (c) have a collective coupling strength 
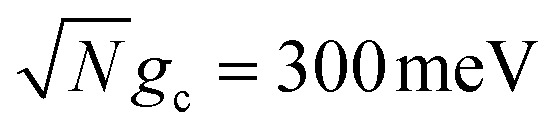
, while panels (b) and (d) have 
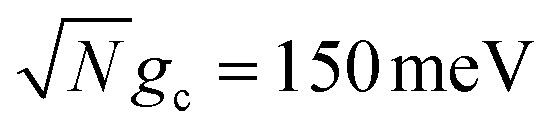
. Simulations were performed with *N* = 1000 molecules, *λ*_A_ = 100 meV, *V*_DA_ = 10 meV, and Δ*G* − *λ*_D_ = 300 meV uphill.

Fig. 2 presents the influence of dark state transfer and cavity loss on PMET population dynamics for an uphill reaction with *N* = 1000. The reaction inside the cavity is initiated as a FC excitation to the resonant |+〉 state. The ET parameters are *λ*_A_ = 100 meV, *V*_DA_ = 10 meV, and Δ*G* − *λ*_D_ = 300 meV uphill at room temperature *T* = 300 K. In this parameter regime, the forward rate constant outside the cavity is very small (*k*^outside^_f_ ≈ 10^−6^ ps^−1^) due to the uphill Δ*G* that is more than 10 times larger than *k*_B_*T*. As a result, population that resides in the dark states (or |**G**, 0〉) will not appreciably transfer to the acceptor states on the plotted timescales (up to 1000 ps). Any visible accumulation of acceptor population must come directly from the UP state due to its more favorable Δ*G*_+**A**_*j*__. Similarly, the backward rate from the acceptor to the donor states outside the cavity is also very small (for nuclear distributions in the FC region). Notably, in contrast to the forward transfer direction, the total backward rate from the acceptor states to the donor manifold is directly influenced by the density of states in the donor manifold. Consequentially, the contribution of the acceptor-to-dark transfer to the total backward rate is (*N* − 1)/(*N* + 1) ≈ 1 which means population transferred to the acceptor states will not appreciably transfer backward to the donor manifold on the plotted timescales. The acceptor population dynamics in Fig. 2 can thus be understood as a result of two competing pathways: the UP to acceptor transfer which appreciably accumulates acceptor population, and the UP to dark or UP to |**G**, 0〉 transfer which becomes effectively “trapped” in the dark or |**G**, 0〉 states and does not lead to appreciable acceptor state population.

Fig. 2a and b show the acceptor population dynamics for varying *λ*_D_ from 0 to 50 meV and for collective coupling strengths 
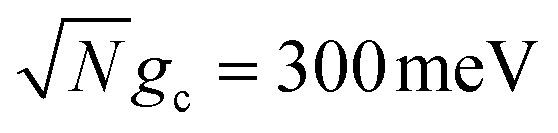
 and 
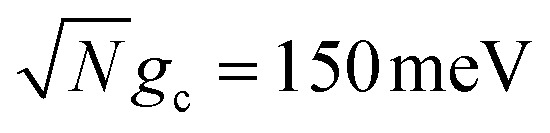
, respectively, with *Γ* = *V*_l_ = 0. Note that by setting *V*_l_ = 0, the CW laser excitation is not active in these simulations. Instead, the system is initially prepared as a Franck–Condon excitation where the initial state is the |+〉 state. The bare driving force Δ*G* is adjusted such that Δ*G* − *λ*_D_ = 300 meV is fixed for all values of *λ*_D_ in order to put the forward rates from the UP to acceptor states on equal footing (*i.e.* so that the driving force in eqn (23) is equal for all values of *λ*_D_ aside from the collective coupling 
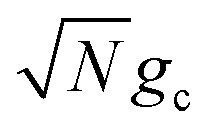
 effect). The primary effect of increasing *λ*_D_ in this case is an increase in the transfer rate from the UP to the dark states. One perspective to understand this effect is that increasing *λ*_D_ increases the coupling strength of the phonon bath that transfers population between the UP and the dark states.^80,88,99^ Another perspective is that increasing *λ*_D_ increases the spectral linewidth of the donor states (*i.e.* adds disorder to the donor energies) which increases the spectral overlap between the UP and dark states resulting in increased transfer between these states. For a Gaussian sampling of nuclear configurations in the FC region, this spectral disorder has a standard deviation of 
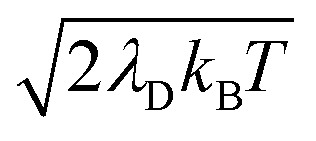
. While the dark states can also transfer to the UP (and LP), this rate scales as 1/*N* which becomes negligible in the collective limit.^80^

The acceptor population dynamics in Fig. 2a do not show a strong dependence on *λ*_D_ ≤ 50 meV. For a collective coupling strength of 
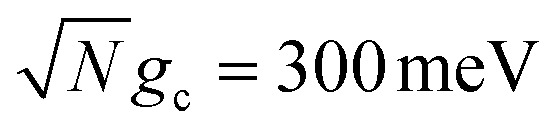
, the reaction is almost barrier-less due to the favorable driving force Δ*G*_+**A**_*j*__ = 0. The initial UP population can thus fully transfer to the acceptor states in only a few ps while the transfer rate from the UP to the dark states is suppressed by the large energy gap between these states even for *λ*_D_ = 50. On the other hand, in Fig. 2b, there is a strong dependence on *λ*_D_ due to the smaller collective coupling strength of 
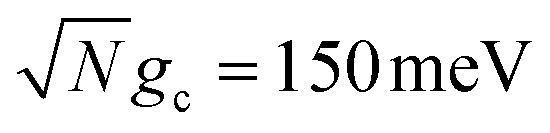
 which both reduces the UP to acceptor rate constant and allows for a significant spectral overlap between the UP and dark states. As a result, a modest *λ*_D_ = 10 meV causes most population to transfer to the dark states before it can transfer to the acceptor states and *λ*_D_ = 50 meV prevents any visible accumulation of acceptor population on these timescales. Thus the magnitude of effect that dark states have on suppressing UP-to-acceptor PMET depends on the competing rates of the UP-to-acceptor pathway and the UP-to-dark pathway, the latter of which is very sensitive to the spectral gap (or lack thereof) between the UP and dark states. As a result, the presence of dark states in PMET must be seriously considered when the timescale of the UP-to-acceptor transfer is similar to or longer than the timescale of UP-to-dark transfer.^80^ Similar consequences of dark state transfer have also been described in other related works on cavity-modified photochemistry.^31,111^

Fig. 2c and d show the acceptor population dynamics for varying cavity loss rates with quality factors *Q* ≡ *ω*_c_/*Γ* ranging from lossless *Q* = ∞ (*Γ* = 0) down to *Q* = 10 (*Γ* = 300 meV) and for collective coupling strengths 
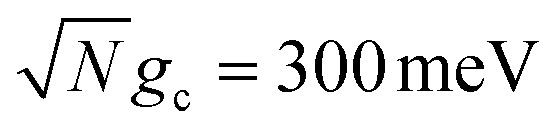
 and 
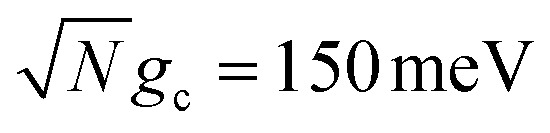
, respectively, with *λ*_D_ = *V*_l_ = 0. The effect of increasing *Γ* in this case is an increase in the transfer rate from the UP to the |**G**, 0〉 state. Due to *V*_l_ = 0, once population is transferred to |**G**, 0〉, it remains trapped there as there are no transfer pathways from |**G**, 0〉 to other states. For *λ*_D_ = 0, the resonant UP state experiences cavity loss at a rate of *Γ*/2 due to the UP possessing half photonic character. In contrast to the dark state transfer rate dependence on *λ*_D_, the cavity loss rate that the UP experiences in this model is not strongly dependent on the energy of the UP or the energy gap between the UP and the dark states. As a result, the acceptor population dynamics in Fig. 2c are visibly suppressed across the entire range of finite *Q*, even for *Q* = 100 000, which corresponds to a minuscule *Γ* = 0.03 meV. The accumulated acceptor population steadily decreases with lower *Q* and higher *Γ* until there is no visible acceptor accumulation for *Q* = 10. In contrast to Fig. 2a, the dynamics in Fig. 2c are not protected from cavity loss by the large collective coupling of the UP. The acceptor population dynamics in Fig. 2d are similarly impacted by lower *Q* factors with decreasing accumulated acceptor population as *Q* is decreased. Due to the lack of energy dependence on the UP cavity loss rate, the difference in the amount of suppression observed between Fig. 2c and d is not as large as that between Fig. 2a and b. Overall, the presence of cavity loss in PMET must be seriously considered when the timescale of the UP-to-acceptor transfer is similar to or longer than the timescale of cavity loss, which is the case for many realistic strongly-coupled molecule–cavity designs^67–70^ whose *Q* are often less than *Q* = 1000, and this cavity loss effect cannot be easily protected against by increasing the collective coupling strength. Similar consequences of cavity loss have also been described in other related works on cavity-modified photochemistry.^58,96^

**Fig. 3:**
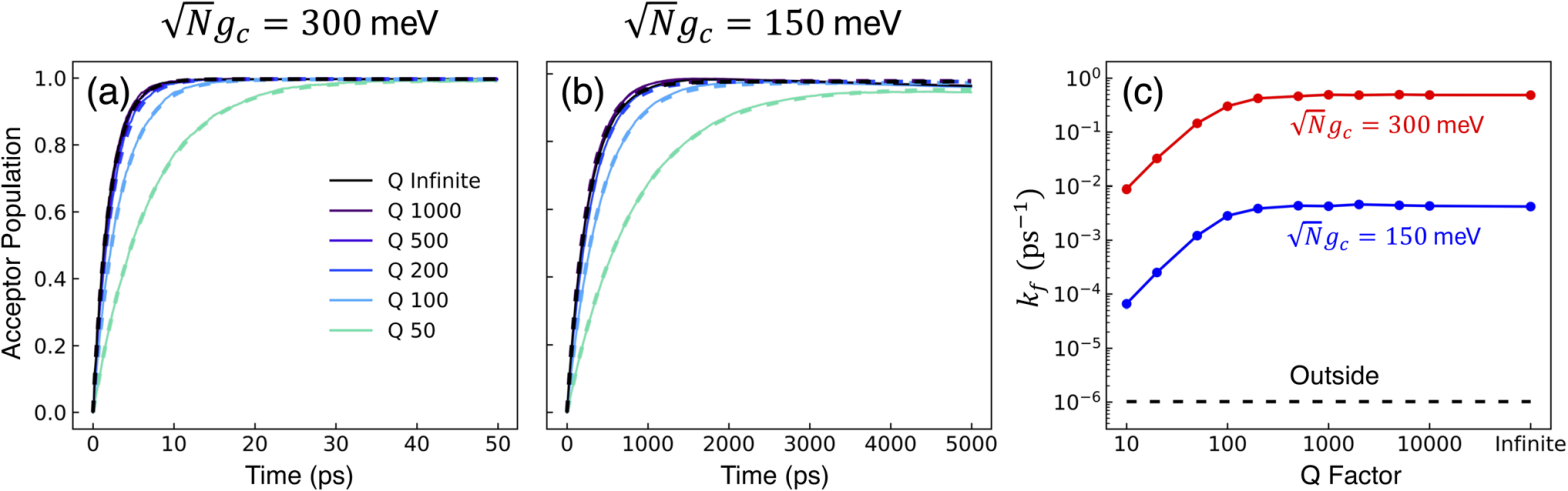
Collective rate enhancement with CW laser driving to the UP in the presence of cavity loss. Panels (a) and (b) are acceptor populations with varying *Q* and laser driving strength of *V*_l_ = 10 meV. Panel (a) has a collective coupling strength 
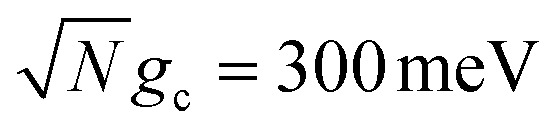
 while panel (b) has 
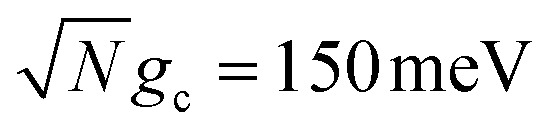
. The solid lines are the propagated populations and the dashed lines are the populations based on the fitted rate constants. Panel (c) shows the forward reaction rate constants fitted from the propagated populations in (a) (red) and (b) (blue) as a function of *Q*. The forward rate constant outside the cavity is shown in black. Simulations were performed with *N* = 1000 molecules, *λ*_A_ = 100 meV, *V*_DA_ = 10 meV, and Δ*G* = 300 meV uphill.

Fig. 3 presents the influence of CW laser driving and cavity loss on PMET population dynamics and rate constants for an uphill reaction with *N* = 1000. The ET parameters are the same as those used in Fig. 2 and *λ*_D_ = 0. The laser pumping strength was set to *V*_l_ = 10 meV and the laser frequency was tuned to 
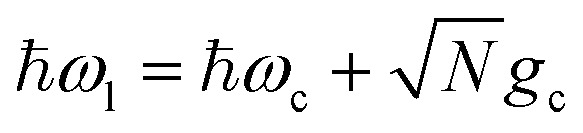
 which is the energy difference between the resonant |+〉 state and |**G**, 0〉 in the FC region. Isolated from other dynamics, this CW laser will coherently drive population between |**G**, 0〉 and the UP due to the photonic character of the UP. Instead of an FC excitation, the reaction inside the cavity is initiated in the |**G**, 0〉 state, which will coherently pump to the UP state and then experience transfer to the acceptor states. The LP state, while also possessing half photonic character, is significantly detuned from *ℏω*_l_ in this case and does not experience any appreciable pumping from |**G**, 0〉.

Fig. 3a and b show the propagated and fitted acceptor population dynamics in the presence of CW laser driving to the UP for varying cavity loss rates from *Q* = 50 to *Q* = ∞ and for collective coupling strengths 
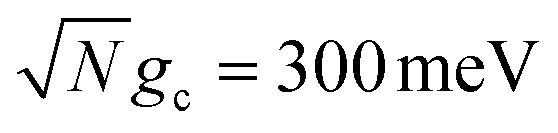
 and 
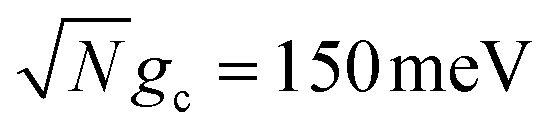
, respectively. As *Q* is decreased in Fig. 3a, the propagated acceptor population (solid lines) accumulates slower yet still approaches *ρ*_{**A**}_ = 1 during the 50 ps of propagation. This stands in sharp contrast to Fig. 2c where lower *Q* factors significantly reduced the plateau acceptor populations. This difference is due to the CW laser in Fig. 3a, which allows for population that was trapped in |**G**, 0〉 due to cavity loss to be repopulated into the UP and continue transfer to the acceptor states. Fig. 3b shows a similar dependence on *Q* where the plateau acceptor populations are much larger than those in Fig. 2d for finite *Q*.

While these dynamics in Fig. 3a and b involve multiple non-ET pathways due to cavity loss and laser driving, the fitted acceptor populations (dashed lines, based on the right-hand side of eqn (29)) still show excellent agreement with the propagated populations. This demonstrates that the combined effect of cavity loss and CW laser driving to the UP can be accurately captured by fitting effective forward *k*_f_ and backward *k*_b_ rate constants to the acceptor population dynamics. These effective rate constants will change based on the cavity loss *Γ* and laser pumping *V*_l_ even though the bare rate constants (such as eqn (28)) between the UP and the acceptor states are unchanged by *Γ* and *V*_l_.

Fig. 3c shows the corresponding fitted forward rate constants from the dynamics in Fig. 3a (red) and b (blue) for a range of *Q* ∈ [10, ∞]. The fitted forward rate constant for a single molecule reaction initiated in |G〉 outside the cavity with a CW laser coupling states |G〉 and |D〉 together with *ℏω*_l_ = Δ*E*_D_, *V*_l_ = 10 meV, and the same ET parameters is plotted as a black dashed line for comparison. The fitted rate constants demonstrate that the PMET reaction is enhanced by orders of magnitude relative to outside the cavity, even for *Q* = 10 and 
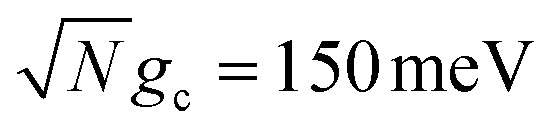
. The shape of the rate constant dependence on *Q* is similar for both 
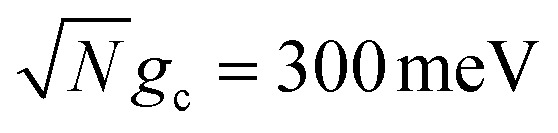
 and 
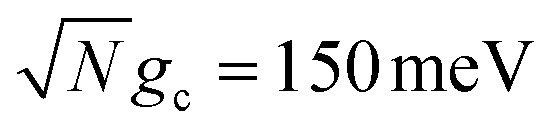
 where the rates are mostly unaffected for *Q* ≥ 500 (*Γ* ≤ 6 meV) and begin to decrease as *Q* decreases below 500. This transition point between affected and unaffected rates along *Q* corresponds to the regime where *ℏΓ* ≈ *V*_l_ because for *ℏΓ* ≪ *V*_l_, the laser pumping can rapidly replenish any UP population lost through cavity loss while for *ℏ*Γ ≫ *V*_l_, the UP population is lost faster than the laser pumping can replenish it. In particular, the effective forward rate constant in this case can be approximated as30
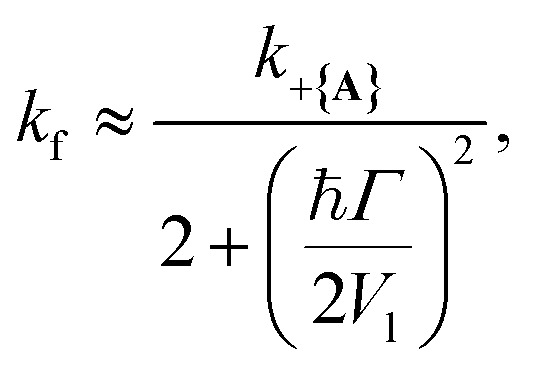
where the effective forward rate constant decreases as the inverse square of *Γ* as *Γ* increases. In addition, a similar dependence on *Q* is expected for different *V*_l_ where the transition point along *Q* is located around *Q* ∼ *ℏω*_c_/*V*_l_. Overall, Fig. 3 demonstrates that the suppression effect of cavity loss as seen in Fig. 2c and d can be alleviated, either partially or entirely, by replenishing the UP population with a CW laser tuned to the UP frequency.

**Fig. 4:**
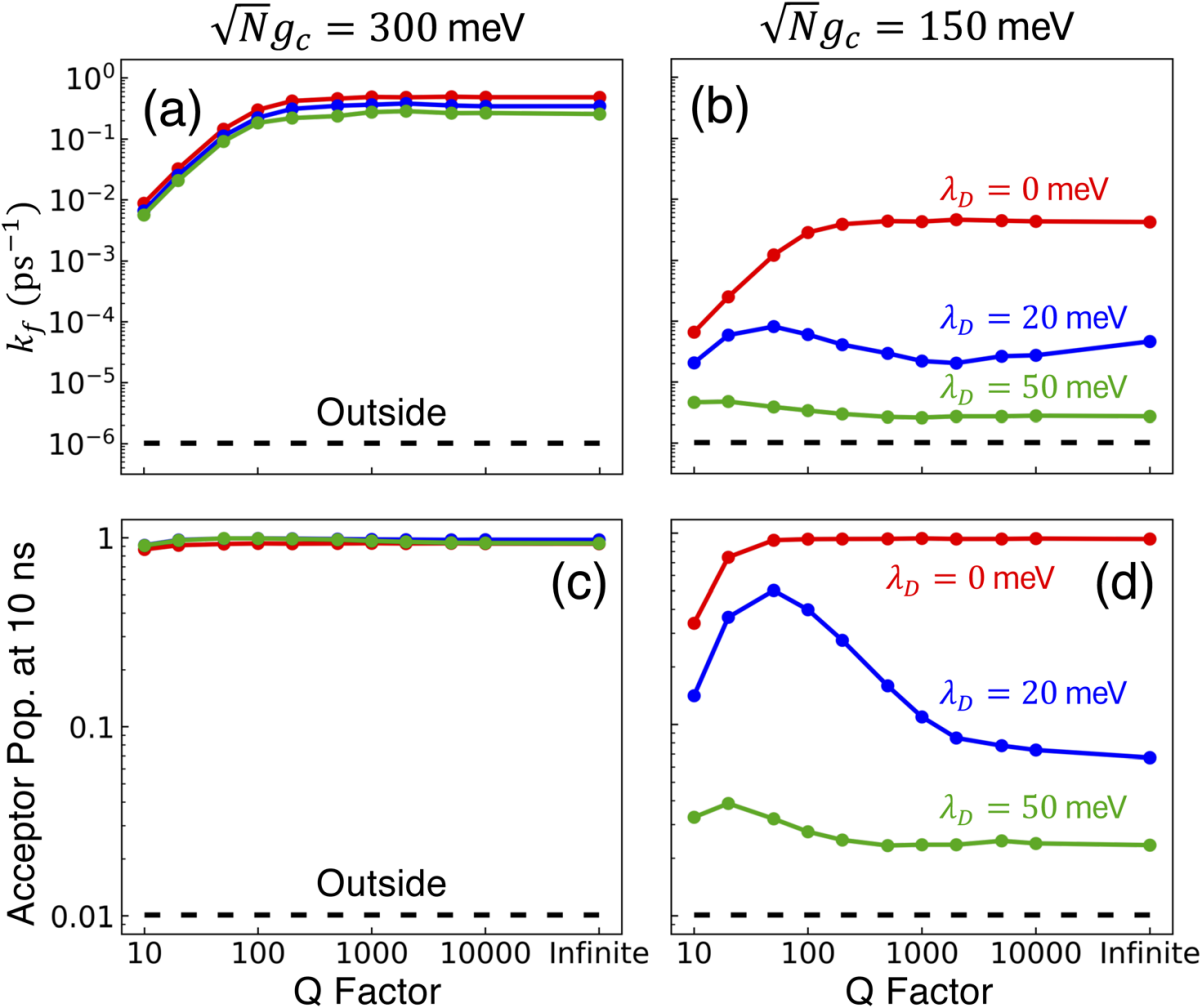
Collective rate enhancement with CW laser driving to the UP in the presence of dark state transfer and cavity loss. Panels (a) and (b) are forward reaction rate constants fitted from propagated populations for varying *Q*, laser driving strength of *V*_l_ = 10 meV, and *λ*_D_ = 0 (red), *λ*_D_ = 20 meV (blue), and *λ*_D_ = 50 meV (green). The forward rate constant outside the cavity is shown in black. Panels (c) and (d) are the acceptor populations after *t* = 10 ns of propagation. The acceptor population after *t* = 10 ns of propagation outside the cavity is shown in black. Panels (a) and (c) have a collective coupling strength 
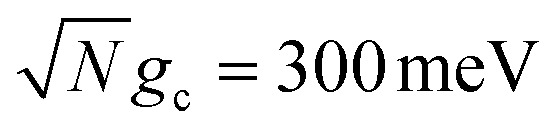
, while panels (b) and (d) have 
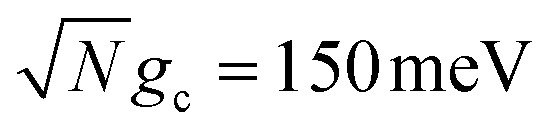
. Simulations were performed with *N* = 1000 molecules, *λ*_A_ = 100 meV, *V*_DA_ = 10 meV, and Δ*G* − *λ*_D_ = 300 meV uphill. The peaks in rate in (b) and peaks in population in (d) for *λ*_D_ = 20 meV and *λ*_D_ = 50 meV are due to the disordered dark states possessing photonic character and experiencing cavity loss which is then pumped back to the UP.

Fig. 4 presents the influence of dark state transfer in addition to CW laser driving and cavity loss on PMET population dynamics and rate constants for an uphill reaction with *N* = 1000. The ET and CW laser parameters are the same as those used in Fig. 3. Simulations were performed for *λ*_D_ = 0 (red), *λ*_D_ = 20 meV (blue), and *λ*_D_ = 50 meV (green) over a range of *Q* ∈ [10, ∞]. The simulations were propagated for *t* = 10 ns because the reaction rates between the dark donor and acceptor states begin to appreciably affect the dynamics around this time and, more broadly, additional dynamics pathways such as non-radiative molecular decay begin to become relevant on these timescales.

Fig. 4a and b show the fitted forward rate constants from the propagated acceptor populations for collective coupling strengths 
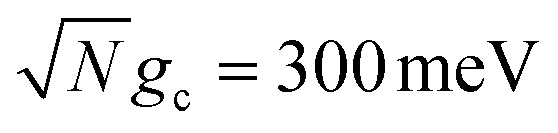
 and 
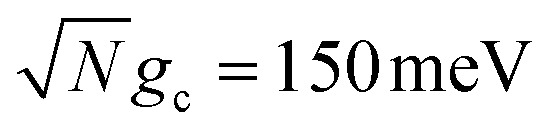
, respectively. For the larger collective coupling simulations in Fig. 4a, the fitted rate constants are only slightly affected by *λ*_D_, where the rates decreased slightly with larger values of *λ*_D_ over the entire range of *Q*. This trend is similar to the dynamics seen in Fig. 2a where the acceptor populations only slightly decreased with increasing *λ*_D_. As discussed before, this is due to the large spectral gap between the UP and the dark states^58^ for 
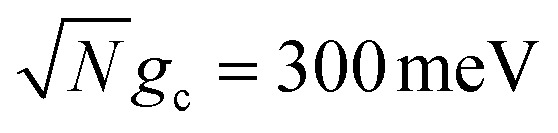
 which greatly reduces the transfer rate from the UP to the dark states compared to smaller collective couplings due to the emergence of the phonon bottleneck effect. As a result, when coherently pumping the UP with a CW laser, the fitted rate constants in Fig. 4a are several orders of magnitude larger than those outside the cavity, even for *λ*_D_ = 50 meV and *Q* = 10.

The fitted rate constants in Fig. 4b have a more complicated dependence on *λ*_D_ than those in Fig. 4a. As demonstrated previously in Fig. 2b, the spectral gap between the UP and dark states for 
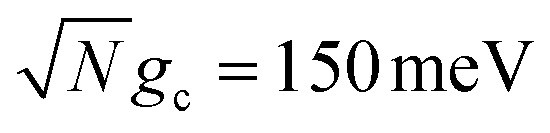
 is small enough that the transfer rate to dark states is significant and the acceptor populations are more sensitive to differences in *λ*_D_. This is observed in the *Q* = ∞ rate constants in Fig. 4b where increasing *λ*_D_ up to 50 meV can reduce the rate constants by up to 3 orders of magnitude compared to *λ*_D_ = 0. Regardless, the fitted rate constants are still significantly larger than those outside the cavity, even for *λ*_D_ = 50 meV. When *Q* is decreased and the cavity loss rate increases, one might expect the fitted rate constants to monotonically decrease as well due to the extra loss channel from the UP to |**G**, 0〉. However, the largest fitted rates for *λ*_D_ = 20 meV and *λ*_D_ = 50 meV are not found at *Q* = ∞ but instead at much smaller, finite values of *Q* (*Q* = 50 for *λ*_D_ = 20 meV and *Q* = 20 for *λ*_D_ = 50 meV). This surprising result can be understood by recognizing that for *λ*_D_ > 0, the dark states are dynamically disordered, which causes them to gain some non-zero photonic character. This photonic character causes the dark states to directly experience cavity loss, which transfers population from the dark states to |**G**, 0〉. With the CW laser tuned to the UP, this population that previously was in the dark states can then be pumped back to the UP, which can then experience enhanced transfer to the acceptor states. In other words, the cavity loss experienced by the disordered dark states allows population previously trapped in these dark states (for *Q* = ∞) to be recycled back into the UP. This mechanism allows for the effective forward rate constant for smaller values of *Q* to be significantly larger than expected based on the *Q* dependence seen in *λ*_D_ = 0 and, in some cases, even be larger than the rate constants for *Q* = ∞. This is a promising result for realizing enhanced PMET experimentally since the *Q* < 1000 attained in many current cavity designs^67–70^ may not be a significant hindrance.

To further examine the interesting PMET dependence on *λ*_D_, Fig. 4c and d show the propagated acceptor populations after *t* = 10 ns of propagation for collective coupling strengths 
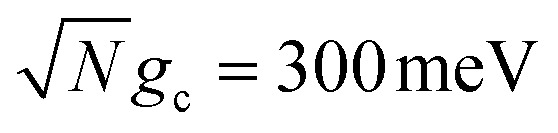
 and 
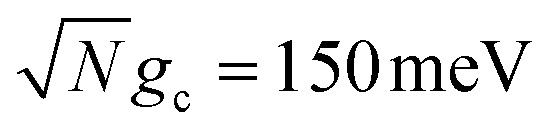
, respectively. These long-time populations are affected not only by the effective forward rate constants shown in Fig. 4a and b but by the effective backward rate constants as well. Simulations with similar forward rate constants but different backward rate constants can have different long-time populations, such that the simulation with a larger backward rate constant can have a smaller long-time acceptor population than the simulation with a smaller backward rate constant. Fig. 4c, like 4a, does not show a strong dependence on *λ*_D_ due to the small rate of transfer to the dark states and, as a result, the long-time acceptor populations are all nearly 1 for all simulated *Q* and *λ*_D_.

On the other hand, Fig. 4d demonstrates the consequence of the disordered dark states cavity loss mechanism seen in Fig. 4b for 
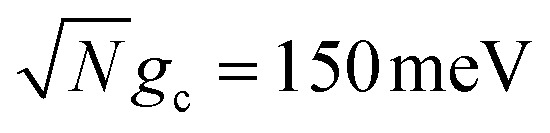
 in an even more striking fashion. Not only are the largest long-time acceptor populations for *λ*_D_ = 20 meV and *λ*_D_ = 50 meV located at smaller values of *Q*, but the smallest long-time acceptor populations are seen at *Q* = ∞, even smaller than those at *Q* = 10. Further, the long-time acceptor population for *λ*_D_ = 20 meV at *Q* = 50 is over 7 times larger than that at *Q* = ∞. While simulations with *Q* = ∞ may have a relatively fast rate of transfer to acceptor states at short times, much of the UP population quickly transfers to and becomes trapped in the dark states for *λ*_D_ > 0 which forbids any appreciable transfer to the acceptor states during the rest of the 10 ns of propagation (we assume the dark exciton lifetime will be longer than this time scale, which is the case for CdSe NPL^67^). When *λ*_D_ > 0 and *Q* is small, on the other hand, the recycling of population from the disordered dark states to the UP allows for acceptor population to continue accumulating for the entire 10 ns of propagation even if the rate of accumulation at the start of the simulation is slower than for *Q* = ∞. This explains why the peaks in long-time acceptor population along *Q* in Fig. 4d are more pronounced than the peaks in forward rate constant in Fig. 4b.

**Fig. 5:**
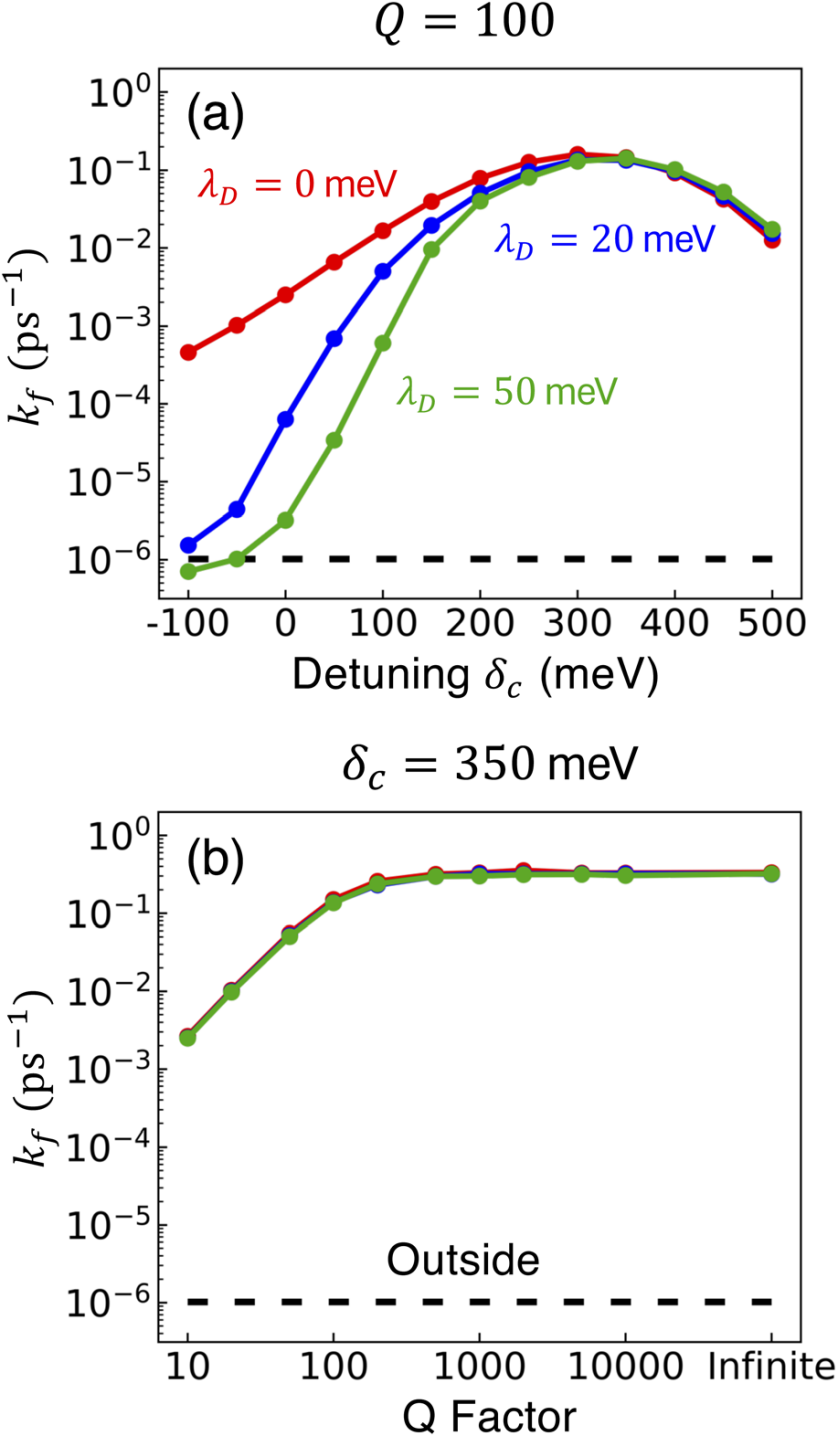
Effect of detuning on collective rate enhancement with CW laser driving to the UP in the presence of dark state transfer and cavity loss. Panel (a) shows the forward reaction rate constants fitted from propagated populations for varying detuning *δ*_c_, fixed *Q* = 100, laser driving strength of *V*_l_ = 10 meV, and *λ*_D_ = 0 (red), *λ*_D_ = 20 meV (blue), and *λ*_D_ = 50 meV (green). Panel (b) is similar to (a) except the detuning is fixed at *δ*_c_ = 350 meV and *Q* is varied. The forward rate constant outside the cavity is shown in black. Simulations were performed with 
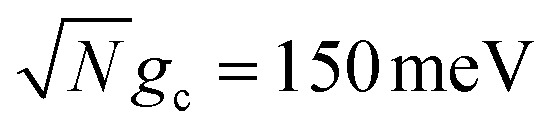
, *N* = 1000 molecules, *λ*_A_ = 100 meV, *V*_DA_ = 10 meV, and Δ*G* − *λ*_D_ = 300 meV uphill.

Fig. 5 presents the influence of cavity detuning in the presence of the CW laser driving, *λ*_D_-induced transition to dark states, and cavity loss on PMET fitted rate constants for an uphill reaction with *N* = 1000. The ET and CW laser parameters are the same as those used in Fig. 3 and 4. Simulations were performed for *λ*_D_ = 0 (red), *λ*_D_ = 20 meV (blue), and *λ*_D_ = 50 meV (green) over *t* = 10 ns with collective coupling 
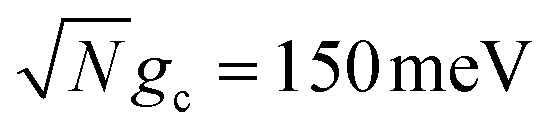
.

Fig. 5a shows the fitted forward rate constants from the propagated acceptor populations with a fixed *Q* = 100 and detuning ranging over *δ*_c_ ∈ [−100, 500] meV. For positive detunings, the rate constant increases up until *δ*_c_ ≈ 350 meV and begins to decrease for larger detunings. The rate constant is maximized near *δ*_c_ ≈ 350 meV because the shoulder of the exponential in eqn (28) is nearly 0 (Δ*G* − *λ*_D_ − (*δ*_c_ + *Ω*_R_)/2 + *λ*_A_ ≈ 0) for this detuning which can be considered a nearly barrier-less transfer from the UP to the acceptor states. While there is some rate reduction at positive detunings due to the decrease of UP matter character sin^2^ *Φ*, this reduction is only sin^2^ *Φ* (*δ*_c_ = 350 meV)/ sin^2^ *Φ* (*δ*_c_ = 0 meV) ≈ 0.24 which is much less than the several orders of magnitude increase of rate due to the more favorable driving force around *δ*_c_ ≈ 350 meV. The rate constant begins to decrease for detunings larger than *δ*_c_ ≈ 350 meV due to both the further reduction of matter character and a driving force that is now in the inverted Marcus regime. For negative detunings, the rate constants decrease for all *λ*_D_, which is expected based on eqn (28) because the UP energy is decreased, which makes the driving force larger and the reaction from the UP to the acceptors slower.

Perhaps the most notable observation from Fig. 5a is that the effective rate constants for all three values of *λ*_D_ become nearly identical for larger positive detunings. This is in stark contrast to the negative detunings, where the non-zero *λ*_D_ rate constants are orders of magnitude smaller than the *λ*_D_ = 0 rate constant. The reasons for this effect at large detunings are similar to the reasons why the effective rate constants for the 
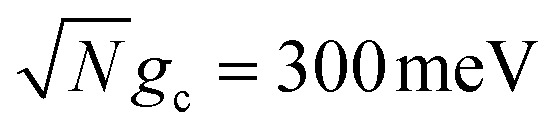
 case in Fig. 4a are nearly the same for all three *λ*_D_ values. The large positive detunings increase the spectral gap between the UP and the dark states, which significantly decreases the transfer rate from the UP to the dark states. In addition, the decrease of UP matter character at positive detunings also decreases the transfer rate from the UP to the dark states since that rate is proportional to the UP matter character. This significantly decreased transfer rate from the UP to the dark states at large positive detunings becomes negligible compared to the increased rate from the UP to the acceptor states, which essentially eliminates the effect of the dark states on the PMET process for large positive detunings. As such, our theoretical work suggests that future experimental work uses positive detunings to (1) enhance the effective driving force of PMET, and at the same time, (2) decrease the transition rate from UP to the dark states.

Fig. 5b shows the fitted forward rate constants from the propagated acceptor populations with a fixed *δ*_c_ = 350 meV and *Q* ranging over *Q* ∈ [10, ∞]. The rate constant's lack of dependence on *λ*_D_ that was seen in Fig. 5a for *Q* = 100 is also seen in Fig. 5b across all values of *Q*. This further supports the idea that the dark states do not significantly impact the overall PMET process from the UP to the acceptor states with large positive detunings. The dependence of the rate constant across *Q* is similar to that seen in Fig. 3c. Additionally, the rate constants in Fig. 5b (with collective coupling 
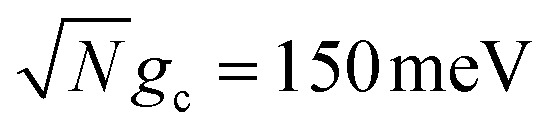
) are almost as large as those rate constants at resonance with 
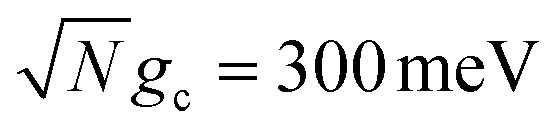
 in Fig. 3c. Notably, the rate constant for *λ*_D_ = 50 meV and *Q* = 10 is 10^3^ times larger than outside the cavity, exemplifying how the cavity can still significantly enhance PMET reactivity even with significant molecular disorder and cavity loss by CW driving to a positively detuned UP state.

## 4 Conclusions

In this work, we theoretically examined enhancing PMET rates in the collective light–matter coupling regime in the presence of dark state transfer and cavity loss. We extended a simple ET model to include collective cavity coupling as well as dark state transfer, cavity loss, and coherent CW laser pumping. We derived the collective reaction coordinate of a PMET reaction between the UP and acceptor states and used it to prove that the driving force from the UP state is decreased by the collective light–matter coupling. We demonstrated the effect of this driving force modification by simulating PMET rate enhancement over a large range of *N* molecules in the Marcus regime and confirmed the excellent agreement between fitted rate constants from simulation and Marcus theory rate constants. We then showed how a CW laser tuned to the UP can partially or entirely avoid these suppressing effects of dark states and cavity loss and allow for orders of magnitude PMET rate enhancement in the collective limit. In particular, we demonstrated that using CW laser driving can effectively reduce the deleterious effect of cavity loss by replenishing the UP populations. Further, a reasonable size of Rabi splitting (larger than the reorganization energy) can reduce the rate of population decay from the UP to the dark states (due to the phonon bottleneck effect^80,99^). Many of the parameters recorded in this work are experimentally relevant to the system of CdSe NPLs coupled to the cavity,^67–70^ and can thus be experimentally tested in the near future. It may be worth mentioning that CW laser driving is often easier to implement than a pulsed laser, and most of photo-driven chemistry is performed under a powerful CW laser source or other continuous narrow-band light source such as a light-emitting diode.

We further considered cavity detuning and found that the effective rate constants can be substantially increased with large positive detuning which also nearly eliminates the suppressing effects of the dark states. Encouragingly, this enhancement can be several orders of magnitude larger than the rate constant outside the cavity even when *λ*_D_ and *Γ* are large. The positive cavity detuning also significantly reduces the magnitude of the population transfer from the UP to the dark states, due to the enlarged energy gap and phonon bottleneck effect. These results demonstrate that significant cavity enhancement may be achievable for uphill ET-like reactions by CW driving to the UP in a cavity that has been positively detuned to optimize the driving force from the UP to the acceptor states and avoid dark state transfer.

These results show that the significant cavity enhancement is enabled by collective coupling to the cavity and depends sensitively on the collective Rabi splitting *Ω*_R_. The collective PMET rate constant depends on 
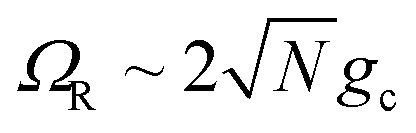
, and thus is collective. Despite individual molecules being weakly coupled to the cavity mode with strength *g*_c_, as long as there are a large enough number of molecules *N* collectively coupled to the cavity, there will be a significant modification of the rate constant. This mechanism relies on the non-local light–matter interactions and local chemical couplings (between donor to acceptor through *V*_DA_). This rate dependence on the collective Rabi splitting in this work could offer insight into the apparent Rabi splitting dependence of existing polariton photochemistry experiments^1,12–15^ such as the rate enhancement observed in ref. 12. Future work includes analyzing the effects of multiple *k* – dispersed cavity modes on the PMET process^68–70,97,101^ as well as investigating both the electron–photon population dynamics and nuclear wave packet dynamics of different photochemical model systems^51,58^ in the collective coupling regime.

## Data availability

Data for this article, including source code and data are available on Github at [https://github.com/ericrkoessler/PMET-LMFE].

## Author contributions

All authors designed the project. E. R. K., A. M., and P. H. derived the analytic expressions of PMET. E. R. K. performed the quantum dynamics simulations. All authors wrote and edited the manuscript.

## Conflicts of interest

The authors declare no conflict of interest.

## Supplementary Material

SC-OLF-D5SC01911G-s001
