# Supplementary material for: Polariton mediated electron transfer under the collective molecule–cavity coupling regime
Source: Chem Sci. 2025 May 19;16(25):11644–58. doi: 10.1039/d5sc01911g (PMC12127852; doi:10.1039/d5sc01911g)
Supplement: SC-016-D5SC01911G-s001 [file SC-016-D5SC01911G-s001.pdf]

Supplementary Information (SI) for Chemical Science.
This journal is © The Royal Society of Chemistry 2025

Supplementary Information: Polariton Mediated Electron Transfer under the Collective Molecule-Cavity Coupling Regime

Eric R. Koessler,[*,†] Arkajit Mandal,[‡] Andrew J. Musser,[¶] Todd D. Krauss,[†,§,‖] and Pengfei Huo[*,†,§,‖]

†*Department of Chemistry, University of Rochester, 120 Trustee Road, Rochester, NY 14627, USA*

‡*Department of Chemistry, Texas A&M University, College Station, TX 77842, USA*

¶*Department of Chemistry and Chemical Biology, Cornell University, Ithaca, NY 14853, USA*

§*Institute of Optics, Hajim School of Engineering, University of Rochester, Rochester, NY 14627, USA*

‖*Center for Coherence and Quantum Optics, University of Rochester, Rochester, NY 14627, USA*

E-mail: ekoessle@ur.rochester.edu; pengfei.huo@rochester.edu

Contents

1 Derivation of the Collective Reaction Coordinate **S2**

2 Langevin Velocity Verlet Algorithm **S5**

3 CW Laser Propagation **S5**

1 Derivation of the Collective Reaction Coordinate

The derivation of the collective reaction coordinate in Eq. 18 of the main text is given below. The derivation relies on the energies of the UP state $|+\rangle$ and the individual acceptor state $|\mathbf{A}_j\rangle$. For $\hat{H}' = \sum_{j=1}^{N}\left(\hat{H}_j - \hat{T}_{\mathrm{S},j} - \hat{H}_{\mathrm{sb},j}\right) + \hat{H}_\mathrm{p} + \hat{H}_\mathrm{mp}$, the UP state $|+\rangle$ energy is

$$E_+(\mathbf{R}) \equiv \langle +|\hat{H}'|+\rangle \tag{S1}$$

$$= \sum_{j'=1}^{N}\left[\frac{1}{2}m_\mathrm{S}\omega_\mathrm{S}^2 R_{j'}^2 + \frac{2\sin^2\Phi}{2N}\left(\Delta E_\mathrm{D} + \frac{1}{2}m_\mathrm{S}\omega_\mathrm{S}^2(R_\mathrm{D}^2 - 2R_{j'}R_\mathrm{D})\right)\right.$$

$$\left. + \frac{2\sin\Phi\cos\Phi\hbar g_\mathrm{c}}{\sqrt{N}}\right] + \frac{2\cos^2\Phi\hbar\omega_\mathrm{c}}{2} \tag{S2}$$

$$= \frac{1}{2}m_\mathrm{S}\omega_\mathrm{S}^2\sum_{j'=1}^{N}\left[R_{j'}^2 - \frac{2\sin^2\Phi}{N}R_{j'}R_\mathrm{D}\right] + \frac{\Delta E_\mathrm{D} + \lambda_\mathrm{D} + \hbar\omega_\mathrm{c} + \Omega_\mathrm{R}}{2} \tag{S3}$$

and the acceptor state $|\mathbf{A}_j\rangle$ energy is

$$E_{\mathbf{A}_j}(\mathbf{R}) \equiv \langle \mathbf{A}_j|\hat{H}'|\mathbf{A}_j\rangle \tag{S4}$$

$$= \sum_{j'=1}^{N}\left[\frac{1}{2}m_\mathrm{S}\omega_\mathrm{S}^2 R_{j'}^2 + \frac{1}{2}m_\mathrm{S}\omega_\mathrm{S}^2(R_\mathrm{A}^2 - 2R_jR_\mathrm{A}\delta_{jj'})\right] + \Delta E_\mathrm{A} \tag{S5}$$

$$= \frac{1}{2}m_\mathrm{S}\omega_\mathrm{S}^2\sum_{j'=1}^{N}\left[R_{j'}^2 - 2R_jR_\mathrm{A}\delta_{jj'}\right] + \Delta E_\mathrm{A} + \lambda_\mathrm{A}. \tag{S6}$$

The difference in energies between the acceptor state and the UP state is

$$\Delta E_{+\mathbf{A}_j}(\mathbf{R}) = E_{\mathbf{A}_j}(\mathbf{R}) - E_+(\mathbf{R}) \tag{S7}$$

$$= \frac{1}{2}m_\mathrm{S}\omega_\mathrm{S}^2\sum_{j'=1}^{N}\left[\frac{2\sin^2\Phi}{N}R_{j'}R_\mathrm{D} - 2R_jR_\mathrm{A}\delta_{jj'}\right] + E_\mathrm{A} + \lambda_\mathrm{A} - \frac{\Delta E_\mathrm{D} + \lambda_\mathrm{D} + \hbar\omega_\mathrm{c} + \Omega_\mathrm{R}}{2} \tag{S8}$$

$$= \frac{2\sin^2\Phi\lambda_\mathrm{D}}{NR_\mathrm{D}}\left[\sum_{j'\neq j}R_{j'} - \left(\frac{NR_\mathrm{A}}{\sin^2\Phi R_\mathrm{D}} - 1\right)R_j\right] + \Delta E^{\mathbf{0}}_{+\mathbf{A}_j}. \tag{S9}$$

Fixing the energy difference as $X = \Delta E_{+\mathbf{A}_j}(\mathbf{R})$ yields the following constraint

$$\left(\frac{NR_{\mathrm{A}}}{\sin^2\Phi R_{\mathrm{D}}} - 1\right) R_j - \sum_{j'\neq j} R_{j'} = \frac{NR_{\mathrm{D}}}{2\sin^2\Phi\lambda_{\mathrm{D}}}\left(\Delta E^{\mathbf{0}}_{+\mathbf{A}_j} - X\right) \tag{S10}$$

The collective reaction coordinate is the nuclear configuration that minimizes $E_+(\mathbf{R})$ under the constraint in Eq. S10. For convenience, we rewrite these equations in a condensed notation such that the UP energy is expressed as

$$E_+(\mathbf{R}) = \alpha_1 \sum_{j'=1}^{N} R_{j'}^2 - \alpha_2 \sum_{j'=1}^{N} R_{j'} + \alpha_3 \tag{S11}$$

and the constraint is expressed as

$$\alpha_4 R_j - \sum_{j'\neq j} R_{j'} = \alpha_5 \tag{S12}$$

where the condensed constants are

$$\alpha_1 = \frac{1}{2} m_{\mathrm{S}} \omega_{\mathrm{S}}^2 \tag{S13}$$

$$\alpha_2 = \frac{2\sin^2\Phi\lambda_{\mathrm{D}}}{NR_{\mathrm{D}}} \tag{S14}$$

$$\alpha_3 = \frac{\Delta E_{\mathrm{D}} + \lambda_{\mathrm{D}} + \hbar\omega_{\mathrm{c}} + \Omega_{\mathrm{R}}}{2} \tag{S15}$$

$$\alpha_4 = \frac{NR_{\mathrm{A}}}{\sin^2\Phi R_{\mathrm{D}}} - 1 \tag{S16}$$

$$\alpha_5 = \frac{NR_{\mathrm{D}}}{2\sin^2\Phi\lambda_{\mathrm{D}}}\left(\Delta E^{\mathbf{0}}_{+\mathbf{A}_j} - X\right) \tag{S17}$$

The constraint in Eq. S12 can be rewritten as

$$R_j = \frac{\alpha_5 + \sum_{j'\neq j} R_{j'}}{\alpha_4} \tag{S18}$$

which allows the R_j coordinate to be eliminated from the minimization along the other $N-1$ coordinates $\{R_{j'\neq j}\}$. Plugging in Eq. S18 into Eq. S11 gives the UP state energy along $\{R_{j'\neq j}\}$

$$E_+(\{R_{j'\neq j}\}) = \alpha_1 \left[\frac{\alpha_5^2 + 2\alpha_5 \sum_{j'\neq j} R_{j'} + \left(\sum_{j'\neq j} R_{j'}\right)^2}{\alpha_4^2} + \sum_{j'\neq j} R_{j'}^2 \right] - \alpha_2 \left[\frac{\alpha_5 + \sum_{j'\neq j} R_{j'}}{\alpha_4} + \sum_{j'\neq j} R_{j'} \right] + \alpha_3 \tag{S19}$$

An unconstrained minimization can now be performed on Eq. S19 where candidate global minimum configurations can be found by setting all $N-1$ partial derivatives to 0 as follows

$$\frac{\partial}{\partial R_{j''}} E_+(\{R_{j'\neq j}\}) = \frac{2\alpha_1\alpha_5 - \alpha_2\alpha_4 - \alpha_2\alpha_4^2}{\alpha_4^2} + \frac{2\alpha_1 \sum_{j'\neq j} R_{j'}}{\alpha_4^2} + 2\alpha_1 R_{j''} \tag{S20}$$

$$= 0 \tag{S21}$$

$$R_{j''} = \frac{\alpha_2\alpha_4 + \alpha_2\alpha_4^2 - 2\alpha_1\alpha_5 - 2\alpha_1 \sum_{j'\neq j} R_{j'}}{2\alpha_1\alpha_4^2} \tag{S22}$$

The equation for $R_{j''}$ in Eq. S22 is identical for all $j'' \neq j$ thus the set of $N-1$ equations can be reduced down to a single equation of a single coordinate $R_{j'}$

$$R_{j'} = \frac{\alpha_2\alpha_4 + \alpha_2\alpha_4^2 - 2\alpha_1\alpha_5 - 2\alpha_1(N-1)R_{j'}}{2\alpha_1\alpha_4^2} \tag{S23}$$

$$R_{j'} = \frac{\alpha_2\alpha_4(1+\alpha_4) - 2\alpha_1\alpha_5}{2\alpha_1(N-1+\alpha_4^2)} \tag{S24}$$

The solution in Eq. S24 gives the unique global minimum of $E_+(\{R_{j'\neq j}\})$. Plugging this equation into Eq. S18 gives the solution for the jth coordinate

$$R_j = \frac{2\alpha_1\alpha_5(N-1+\alpha_4^2)/\alpha_4 + (N-1)(\alpha_2 + \alpha_2\alpha_4 - 2\alpha_1\alpha_5/\alpha_4)}{2\alpha_1(N-1+\alpha_4^2)} \tag{S25}$$

$$= \frac{\alpha_2(N-1)(1+\alpha_4) + 2\alpha_1\alpha_5\alpha_4}{2\alpha_1(N-1+\alpha_4^2)} \tag{S26}$$

Plugging in Eqs. S13-S17 into Eq. S26 and Eq. S24 recovers the collective reaction coordinate in Eq. 18 in the main text.

2 Langevin Velocity Verlet Algorithm

The nuclear positions $\{R_j\}$ and momenta $\{P_j\}$ are propagated using a Langevin-modified velocity Verlet approach.[1,2] For a propagation from time t to time $t + \mathrm{dt_N}$, the position and momentum of molecule j are updated as follows

$$R_j(t + \mathrm{dt_N}) = R_j(t) + \frac{2\mathrm{dt_N}}{2m_\mathrm{S} + \mathrm{dt_N}\zeta}\left(P_j(t) + \frac{\mathrm{dt_N}F_j(t)}{2} + \sqrt{\frac{\mathrm{dt_N}\zeta}{2\beta}}\eta_j\right), \tag{S27}$$

$$\begin{aligned} P_j(t + \mathrm{dt_N}) = P_j(t) &+ \frac{\mathrm{dt_N}\Big(F_j(t) + F_j(t + \mathrm{dt_N})\Big)}{2} \\ &+ \frac{2\mathrm{dt_N}\zeta}{2m_\mathrm{S} + \mathrm{dt_N}\zeta}\left(-P_j(t) - \frac{\mathrm{dt_N}F_j(t)}{2} + \frac{2m_\mathrm{S}}{\mathrm{dt_N}\zeta}\sqrt{\frac{\mathrm{dt_N}\zeta}{2\beta}}\eta_j\right), \end{aligned} \tag{S28}$$

where η_j is a normally distributed random number with mean 0 and unit variance that is independently sampled for each molecule at each timestep, and $F_j(t)$ is the mean-field Ehrenfest force on molecule j evaluated at nuclear position $\mathbf{R}(t)$. Note that the same η_j is used for the position and momenta updates of molecule j for a given timestep without resampling between the position and momenta updates.

3 CW Laser Propagation

For the propagation of the quantum subsystem state $|\Psi(t)\rangle = c_{\mathbf{G}0}(t)|\mathbf{G}, 0\rangle + c_{\mathbf{G}1}(t)|\mathbf{G}, 1\rangle + \sum_{j=1}^{N} c_{\mathbf{D}_j 0}(t)|\mathbf{D}_j, 0\rangle + c_{\mathbf{A}_j 0}(t)|\mathbf{A}_j, 0\rangle$, where $c_i(t) = \langle i|\Psi(t)\rangle$ is the wavefunction expansion coefficient for diabatic state i, the propagation due to the CW laser driving is separated

from the propagation of the other Hamiltonian dynamics through a Trotter decomposition

$$|\Psi(t+\mathrm{dt_E})\rangle = e^{-i\hat{H}\mathrm{dt_E}/\hbar}|\Psi(t)\rangle, \tag{S29}$$

$$\approx e^{-i(\hat{H}-\hat{H}_\mathrm{l})\mathrm{dt_E}/\hbar}e^{-i\hat{H}_\mathrm{l}\mathrm{dt_E}/\hbar}|\Psi(t)\rangle. \tag{S30}$$

The propagation of the state under CW laser driving $e^{-i\hat{H}_\mathrm{l}\mathrm{dt_E}/\hbar}|\Psi(t)\rangle$ can be performed using the exact analytic solution of the dynamics of the two driven states $c_{\mathbf{G}0}(t)$ and $c_{\mathbf{G}1}(t)$. For a propagation from time t to time $t+\mathrm{dt_E}$, the driven wavefunction coefficients are updated as follows

$$c_{\mathbf{G}0}(t+\mathrm{dt_E}) = \frac{e^{-i(\Omega_\mathrm{l}-\omega_\mathrm{l}-\omega_\mathrm{c})\mathrm{dt_E}/2}}{2\Omega_\mathrm{l}} \times \left(-\frac{2V_\mathrm{l}}{\hbar}e^{i\omega_\mathrm{l}t}(e^{i\Omega_\mathrm{l}\mathrm{dt_E}}-1)c_{\mathbf{G}1}(t) + (\Omega_\mathrm{l}+\omega_\mathrm{l}-\omega_\mathrm{c}+e^{i\Omega_\mathrm{l}\mathrm{dt_E}}(\Omega_\mathrm{l}-\omega_\mathrm{l}+\omega_\mathrm{c}))c_{\mathbf{G}0}(t)\right), \tag{S31}$$

$$c_{\mathbf{G}1}(t+\mathrm{dt_E}) = \frac{e^{-i(\Omega_\mathrm{l}+\omega_\mathrm{l}-\omega_\mathrm{c})\mathrm{dt_E}/2}}{2\Omega_\mathrm{l}} \times \left(-\frac{2V_\mathrm{l}}{\hbar}e^{-i\omega_\mathrm{l}t}(e^{i\Omega_\mathrm{l}\mathrm{dt_E}}-1)c_{\mathbf{G}0}(t) + (\Omega_\mathrm{l}-\omega_\mathrm{l}+\omega_\mathrm{c}+e^{i\Omega_\mathrm{l}\mathrm{dt_E}}(\Omega_\mathrm{l}+\omega_\mathrm{l}-\omega_\mathrm{c}))c_{\mathbf{G}1}(t)\right), \tag{S32}$$

where $\Omega_\mathrm{l} = \sqrt{\left(\frac{2V_\mathrm{l}}{\hbar}\right)^2 + (\omega_\mathrm{l}-\omega_\mathrm{c})^2}$ is an effective laser Rabi frequency. Note that for computational convenience, the energies of the quantum states have been reduced by the photon energy $\hbar\omega_\mathrm{c}$ such that the average energy and phase accumulation of the excited states are reduced. The above split propagation procedure allows for a larger electronic timestep $\mathrm{dt_E}$ to be used compared to a single combined propagation of $\hat{H}$ since the excited state energy differences and couplings in $\hat{H}-\hat{H}_\mathrm{l}$ are significantly smaller than the laser frequency energy $\hbar\omega_\mathrm{l}$ and photon energy $\hbar\omega_\mathrm{c}$. Additionally, a Verlet-like symplectic approach[3] can be used to propagate the $\hat{H}-\hat{H}_\mathrm{l}$ dynamics which significantly reduces the accumulated numerical error over long propagation times compared to a non-symplectic approach like RK4. This

Verlet-like symplectic approach is useful since $\hat{H} - \hat{H}_l$ is slowly varying in time; the rapid time fluctuations of $\hat{H}_l$ would otherwise significantly reduce the accuracy of such an approach.

References

(1) Grønbech-Jensen, N.; Farago, O. A simple and effective Verlet-type algorithm for simulating Langevin dynamics. *Molecular Physics* **2013**, *111*, 983–991.

(2) Paterlini, M.; Ferguson, D. M. Constant temperature simulations using the Langevin equation with velocity Verlet integration. *Chemical Physics* **1998**, *236*, 243–252.

(3) Tannor, D. *Introduction to Quantum Mechanics: A Time-Dependent Perspective*; Univ. Science Books: Sausalito, 2007; p 316.
